# Characterizing Extracellular Vesicles and Their Diverse RNA Contents

**DOI:** 10.3389/fgene.2020.00700

**Published:** 2020-07-17

**Authors:** Eren M. Veziroglu, George I. Mias

**Affiliations:** ^1^Institute for Quantitative Health Science and Engineering, Michigan State University, East Lansing, MI, United States; ^2^Department of Biomedical Engineering, Michigan State University, East Lansing, MI, United States; ^3^Department of Biochemistry and Molecular Biology, Michigan State University, East Lansing, MI, United States

**Keywords:** extracellular vesicle, exosome, RNA, gene expression, transcriptome, microvesicle, biomarker, characterization

## Abstract

Cells release nanometer-scale, lipid bilayer-enclosed biomolecular packages (extracellular vesicles; EVs) into their surrounding environment. EVs are hypothesized to be intercellular communication agents that regulate physiological states by transporting biomolecules between near and distant cells. The research community has consistently advocated for the importance of RNA contents in EVs by demonstrating that: (1) EV-related RNA contents can be detected in a liquid biopsy, (2) disease states significantly alter EV-related RNA contents, and (3) sensitive and specific liquid biopsies can be implemented in precision medicine settings by measuring EV-derived RNA contents. Furthermore, EVs have medical potential beyond diagnostics. Both natural and engineered EVs are being investigated for therapeutic applications such as regenerative medicine and as drug delivery agents. This review focuses specifically on EV characterization, analysis of their RNA content, and their functional implications. The NIH extracellular RNA communication (ERC) program has catapulted human EV research from an RNA profiling standpoint by standardizing the pipeline for working with EV transcriptomics data, and creating a centralized database for the scientific community. There are currently thousands of RNA-sequencing profiles hosted on the Extracellular RNA Atlas alone (Murillo et al., 2019), encompassing a variety of human biofluid types and health conditions. While a number of significant discoveries have been made through these studies individually, integrative analyses of these data have thus far been limited. A primary focus of the ERC program over the next five years is to bring higher resolution tools to the EV research community so that investigators can isolate and analyze EV sub-populations, and ultimately single EVs sourced from discrete cell types, tissues, and complex biofluids. Higher resolution techniques will be essential for evaluating the roles of circulating EVs at a level which impacts clinical decision making. We expect that advances in microfluidic technologies will drive near-term innovation and discoveries about the diverse RNA contents of EVs. Long-term translation of EV-based RNA profiling into a mainstay medical diagnostic tool will depend upon identifying robust patterns of circulating genetic material that correlate with a change in health status.

## 1. Introduction

Extracellular vesicles (EVs) are secreted, nanometer-scale genetic information carriers found in human biofluids. Aside from EVs, there are a number of other non-vesicular nanoparticles in circulation such as lipoproteins, RNA-binding proteins, and exomeres (Jeppesen et al., 2019). EVs are broadly defined as lipid bilayer enclosed packages of biomolecules released from cells into their surrounding environment, and include particles described as exosomes, ectosomes, microvesicles, oncosomes, and apoptotic bodies, among other names. EVs vary widely in their size (<50 nm to several μm in diameter), chemical compositions, and purported functions depending on how they are formed and the cell types by which they are produced (Théry et al., 2018).

### 1.1. Historical Background

**Figure 1:**
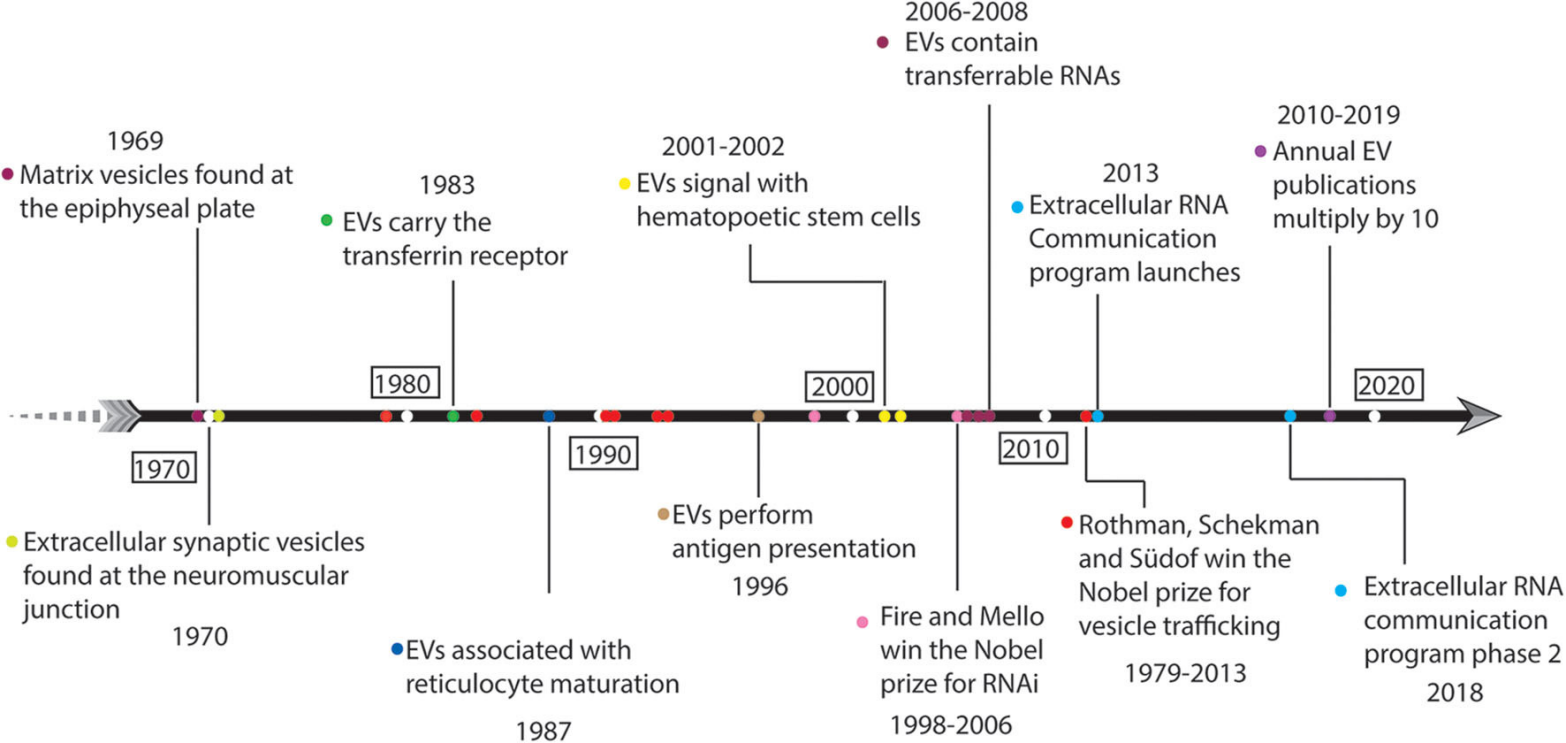
Fifty years of historical landmarks in extracellular vesicle-related research.

EVs in mammalian systems have been recognized in published work for at least 50 years (Figure 1), yet their biological purpose has generally eluded scientific understanding. Mammalian gene expression through EVs and the functional roles thereof were recognized as early as 1969 when H. Clarke Anderson and colleagues identified the association of EVs with epiphyseal cartilage matrix calcification in mice (Anderson, 1969; Ali et al., 1970). Concurrently, Mary Grillo identified EVs in the periaxonal space within the mouse atrium and proposed a model for neuronal signaling which combined merocrine and apocrine secretory processes (Grillo, 1970). There were additional reports describing extra-axonal or extracellular synaptic vesicles at sites of thyroid gland (1963) and arrector pilorum (1965) innervation even before Grillo and Anderson had published. Grillo's findings were criticized at the time as experimental artifact (Dermietzel et al., 1972); however, modern theories now incorporate EVs as a means of neuronal signaling (Basso and Bonetto, 2016; Budnik et al., 2016) and recapitulate her idea that EVs perform signaling functions. EV reports in human biomedical research are cited back as early as 1976. Human erythrocytes treated with a divalent cation ionophore, A23187, exhibited increased membrane diacylglycerol (DAG) content and released EVs enriched in DAG. The A23187-induced EVs accounted for half of the new DAG produced (Allan et al., 1976), giving early evidence that EVs are associated with mechanisms for dealing with cellular stress. In 1983, it was observed that sheep reticulocytes shed their transferrin receptors by releasing EVs during maturation (Pan and Johnstone, 1983). “Exosome” became the term to describe these EVs when Johnstone and colleagues theorized in a later study that EV secretion is a mechanism to remove membrane components that are no longer needed during reticulocyte maturity (Johnstone et al., 1987). Moving forward from Johnstone's studies in the 1980s we see extensive misuse and lack of nomenclature standardization in EV studies, which to this day prevents progress in the research community (Théry et al., 2018). Furthermore, the misinterpretation of the sheep reticulocyte studies led many to believe that EVs were simply a means to dispose of unwanted cellular components. Looking back on this period we can see evidence for EV involvement across global pathways such as intercellular signaling (Grillo, 1970), cellular stress responses (Allan et al., 1976), cell maturation (Pan and Johnstone, 1983), and development (Anderson, 1969; Ali et al., 1970).

Starting around 1996, a series of publications began describing the ability of EVs to elicit complex signaling functions in cellular systems (Figure 1). Raposo et al. used immunoelectron microscopy to observe that B cells from both humans and mice secrete EVs carrying the major histocompatibility complex class-II (MHC-II) molecule. MHC-II restricted T-cell responses were functionally initiated by B cell-derived EVs (Raposo et al., 1996) indicating that EVs can perform specialized cell signaling functions. Raposo et al. were foundational in establishing EVs as intercellular communication agents, and a number of hematology studies followed suit reporting signaling activities associated with EVs, such as enhancing hematopoetic stem cell proliferation, survival, adhesion, and chemotaxis (Janowska-Wieczorek et al., 2001; Baj-Krzyworzeka et al., 2002). The accelerated development and implementation of molecular biology tools to study nucleic acids began elucidating the importance and biological function of EVs. In separate studies, Ratajczak, Valadi, and Skog each showed that EVs contain RNA, and that EV-derived RNAs were transferable to recipient cells (Ratajczak et al., 2006; Valadi et al., 2007; Skog et al., 2008). Interestingly, Valadi et al. found no clear correlation between EV expression and parent cell (Valadi et al., 2007) which is contradictory with later studies (Wei et al., 2017). The combination of the 2006 Nobel Prize being awarded to Fire and Mello for their discovery of RNA interference (Fire et al., 1998), and Valadi et al. establishing the presence of small RNAs including micro-RNAs within EVs (Valadi et al., 2007; Skog et al., 2008) together pushed EV research into the spotlight.

Over the past decade, EV research has continued to rise in prominence (Figure 1). The number of articles listed in the Web of Science database using the search strategy “‘exosome*’ OR ‘microvesicle*’ OR ‘extracellular vesicle*’” has increased approximately 10-fold from 2010 to 2019 (from ~400 to ~5,000^1^). Furthermore, in 2013, Rothman, Schekman, and Südof were awarded a Nobel Prize for elucidating molecular mechanisms of vesicle trafficking within cells. While this award was given for a series of discoveries published between 1979 and 1993 (Novick and Schekman, 1979; Balch et al., 1984; Kaiser and Schekman, 1990; Perin et al., 1990; Hata et al., 1993; Söllner et al., 1993), it indicates the scientific community's valuation of vesicle biology during the 2010s. From these discoveries it became clear that cells dedicate a vast amount of resources and focus toward regulating vesicle traffic. Yet, EV biology is still unclear with regard to active regulation (organized loading) of their contents and secretion. Advances in omics technologies, such as massively-parallel nucleic acid sequencing (Mardis, 2008; McCombie et al., 2019), have enabled a wide range of discovery-based and hypothesis-driven EV research syndicated by the NIH ERC program (Das et al., 2019). EV-derived RNAs are detectable in nearly all human biofluids (Godoy et al., 2018). EVs are associated with development (Bianchi et al., 2014; Robbins, 2017; Takasugi, 2018), circulating tumor DNA (Vagner et al., 2018), insulin resistance and metabolic phenotypes (Shah et al., 2017a), athletic performance (Capomaccio et al., 2013; Shah et al., 2017b; Whitham et al., 2018), cardiovascular disease (Shah et al., 2018), allergic responses (Pua et al., 2019), and calcification (Shapiro et al., 2015; Cui et al., 2016; Hasegawa et al., 2017; Li et al., 2019) among other physiological phenomena. The diversity of extracellular nucleic acids in human biofluids goes beyond endogenous expression, raising the importance of microbiota and dietary sources of RNA. Several reports have shown that RNAs from bacteria, fungi, and other species are of a significant fraction in human plasma and saliva (Wang et al., 2012; Fritz et al., 2016). Current theories suggest that all cell types secrete EVs, and that EVs functionally carry DNA, RNA, protein, and lipid molecules, thereby allowing cells to communicate amongst each other and orchestrate physiological states.

In the remainder of this Review, we first describe our current understanding of EV biogenesis and fates. Then, we focus on experimental approaches for EV separation and concentration and EV characterization. Additionally, we discuss EV composition, and focus on the diverse RNA contents that have been discovered in EVs. Finally, we describe EV physiology and biomedical relevance, and conclude with a summary of current resource databases where EV data are being provided.

## 2. Biogenesis and Fates

EV biogenesis studies fundamentally aim to understand how a cell forms and secretes vesicles. Biogenesis studies can infer from cellular mechanisms how EVs formed by different biogenesis pathways differ with regard to their function, if at all. Biogenesis pathways could differ in the way that they sample membrane-derived cell fractions and display them, thereby acting as a mechanism to specifically communicate internal states. As an example, consider the endogenous vs. exogenous antigen display pathways (Blum et al., 2013). EVs made through different biogenesis pathways could also hypothetically carry and functionally transfer different types of genetic information (Kanada et al., 2015). If EV functions are different based on their biogenesis, then we can ask what mechanisms regulate their production and how those biogenesis pathways can be perturbed. From an RNA standpoint, we are interested in what circulating EV-related RNA implies about the parent cell and the organism state as a whole.

**Figure 2:**
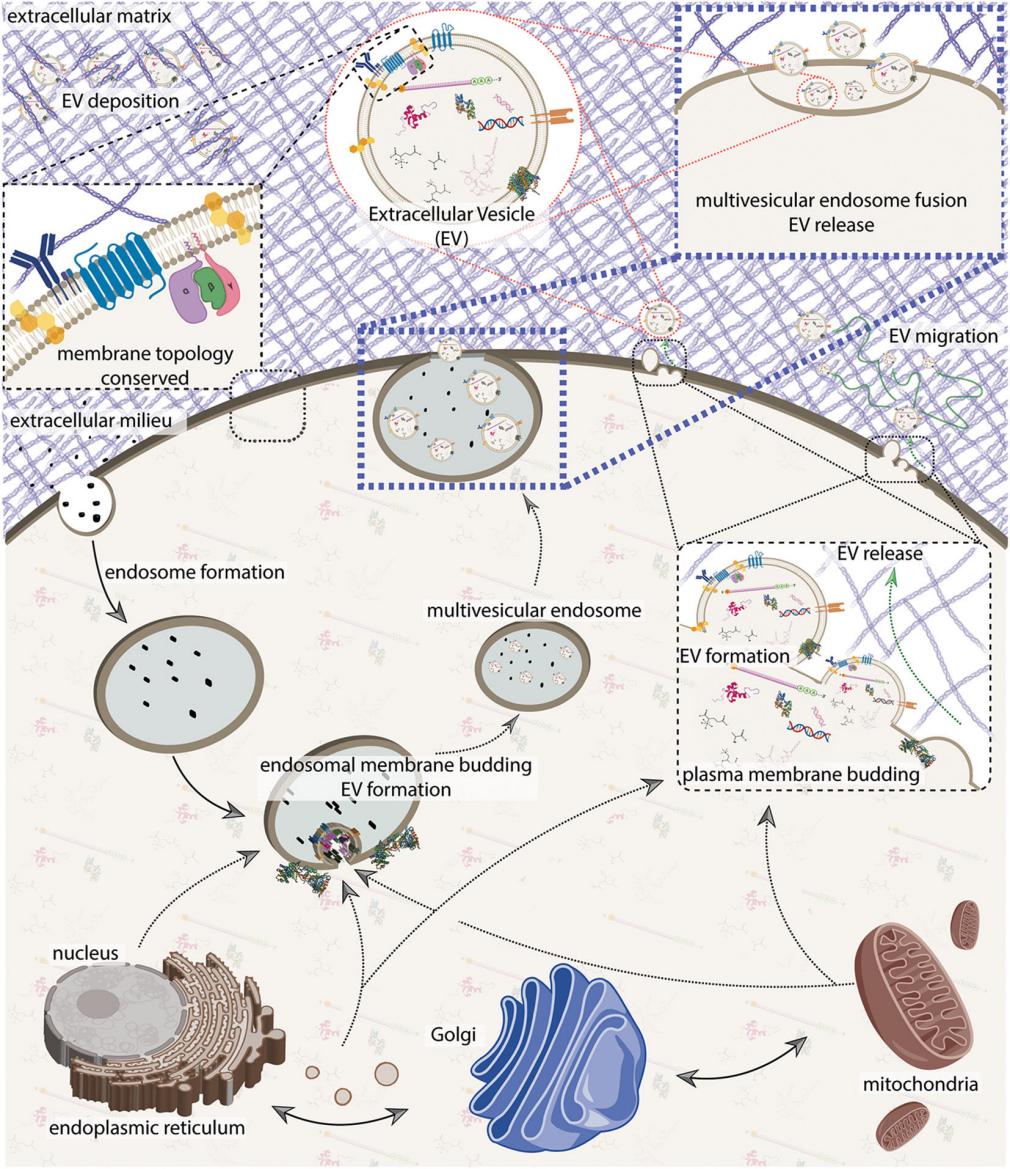
An integrative model of extracellular vesicle (EV) biogenesis. A sample of plasma membrane and extracellular materials is internalized through the endocytic pathway, forming an endosome. The nucleus, endoplasmic reticulum, Golgi, and mitochondria generate an interconnected secretory network that can deliver cellular contents either to the endosome or to the plasma membrane. Secretory machinery localizes at either the endosome or at the plasma membrane and contents are loaded into the nascent EV while membrane budding away from the cytosol occurs. Membrane scission occurs, and at the plasma membrane, EVs are immediately released. In the endosomal pathway, nascent EVs are kept as intralumenal vesicles until the multivesicular endosome fuses with the plasma membrane to release its contents. Cell membrane topology and constituents are generally conserved. On release, EVs can either bind to or navigate through the extracellular milleu which can include matrix proteins. See Figure 4 for more detail on EV composition. The figure was prepared in part using BioRender.com.

There are two predominant EV biogenesis pathways. The first biogenesis pathway buds EVs directly from the plasma membrane, forming what are classically termed microvesicles. The second biogenesis pathway involves intralumenal vesicle release by multivesicular endosome fusion with the plasma membrane, forming what are classically termed exosomes. Due to the MISEV2018 guidelines, and challenges with EV classification as we discuss further below, we refrain from the continued usage of this classic nomenclature (Théry et al., 2018). For recent and detailed reviews covering EV biogenesis and related cell biology (see van Niel et al., 2018; Mathieu et al., 2019). Briefly, EV biogenesis can be thought of in three generalized steps (Figure 2):

1. Membrane components aggregate, and cellular machinery localizes to form a microdomain at the site of the nascent EV.
2. The membrane buds outward, away from the cytosol, and vesicle contents are loaded.
3. The nascent EV membrane is cleaved.

The endosomal biogenesis pathway is distinct from the “direct budding" pathway insofar as the enumerated steps are executed at the late-endosome, vs. at the plasma membrane. The multivesicular endosome then fuses with the plasma membrane, thereby releasing the EVs (Figure 2). Major proteins involved with EV biogenesis include CD63, CD81, CD9, ALIX, TSG101, syntenin, ubiquitin, clathrin, VPS32, VPS4, ERK, PLD, and ARF6 (van Niel et al., 2018; Jeppesen et al., 2019). In both processes, membrane topology is generally conserved (Figure 2), however membrane component flipping can also occur. It is important to note that the distinction between these pathways is becoming less clear as we become more aware of pathway interdependencies and cell specialization (Booth et al., 2006; van Niel et al., 2018), as well as the possibility of additional unrecognized pathways. It can be useful to conceptualize EVs by these two biogenesis pathways, but keep in mind the vast amount of diversity among EVs and the limitations of operating with simplifying models.

The extent to which EV components are actively selected is controversial (Pegtel and Gould, 2019) especially with regard to RNAs (Mateescu et al., 2017). A number of studies argue due to differential RNA, protein, and lipid content of EVs vs. their parent cell that there is selective loading of those EV contents. However, we agree with Pegtel et al. that a number of biophysical factors confound the inference that differential composition implies active selection. Considering an EV volume of 4 × 10^−21^ *m*^3^ (sphere of radius 0.1 μm) vs. a eukaryotic cell of 4 × 10^−15^ *m*^3^ (sphere of radius 10 μm), the volumes are different by a factor of 10^6^ and only certain cellular sub-regions are necessarily sampled by the nature of EV biogenesis requiring a membrane. To this end, microscale sampling of membrane and cytosolic components along with stochastic variance in molecule distribution within the cell, and other biophysical and biochemical factors imply that differential composition of EVs relative to their parents is insufficient to claim organization. Active loading of EV-associated proteins is implied in limited instances, and we suspect they are those identified as biogenesis-related EV biomarkers such as CD63, CD9, CD81, Annexin A1, and TSG101 (Jeppesen et al., 2019). We found strong mechanistic evidence that at least some RNAs are actively loaded into EVs (Pegtel et al., 2010; Cha et al., 2015; Shurtleff et al., 2016, 2017; Teng et al., 2017; Biró et al., 2019; Clancy et al., 2019; Leidal et al., 2020) though we suspect, due to reasons described above, that a significant portion of RNAs are not actively selected for.

A major limitation in the EV field with regard to understanding basic vesicle biology is the disconnection between observing vesicle formation and deeply characterizing them. Since there is currently no clear connection between EVs formed in a specific biogenesis pathway, and measurable characteristics of those EVs, it remains logically challenging to make any claims about EVs produced by one biogenesis pathway vs. another. Consider the following thought experiment which illustrates why it is difficult to make claims about the characteristics of EVs produced through a specific EV biogenesis route. We observe that vesicles formed by direct membrane budding have a mode size of 250 nm while those formed through the endosomal pathway have a mode size of 100 nm. Then, we infer that larger EVs are developed through direct budding and smaller EVs are endosomal in origin. In a separate experiment, we obtain a sample of hematopoietic stem cell-conditioned media, and isolate EVs using a size-exclusion technique that yields >200 nm EV and <200 nm EV fractions. Assume in this case that there are no other extracellular particles aside from EVs and that the size exclusion technique functions perfectly. Now, we add these two EV subpopulations to whole blood. Upon biochemical analysis, we find that whole blood exposed to <200 nm EVs acquired increased stemness, while the sample exposed to >200 nm EVs did not. We conclude that <200 nm EVs can functionally confer stemness, while >200 nm EVs lack this capability. By corollary, endosomal-derived hematopoietic stem cell EVs can functionally confer stemness while direct membrane budded EVs cannot. However, we later invalidate this corollary theorem when we realize that both EV biogenesis pathways produce small (<200 nm) EVs (Booth et al., 2006; Jeppesen et al., 2019). Therefore, studying EV functions by size fractionation is insufficient to link biogenesis mode with function. Here, we use sizing qualities as an example characteristic to describe the challenges of classifying EV subsets by biogenesis pathways; however, the above logic carries to many other experimental inquiries. To this end, a technology which can enable selective study of a single biogenesis pathway will allow for great advancement in our understanding of EV subtypes, though this presents a significant challenge due to the amount of shared cellular machinery between pathways (van Niel et al., 2018; Mathieu et al., 2019). Furthermore, biogenesis pathway interdependencies can produce phenomena such as EVs which biochemically and biophysically resemble an endosomally derived EV that were in fact produced by direct membrane budding (Booth et al., 2006), indicating that cells can specialize to operate beyond a binary classification of EV biogenesis pathways. More subtle protein engineering experiments may be able to tease out the molecular mechanisms related to EV release by different biogenesis pathways and thereby enable discrete EV subtype characterization. For example, one could perturb RAB-dependent EV release by blocking specific ubiqitinylation sites (Song et al., 2016) or sensing (e.g., cholesterol-sensitive) domains (Möbius et al., 2003; Rocha et al., 2009), and then perform deep profiling on EVs released under each circumstance. We are not currently aware of any method to *de novo* select for vesicles from a particular biogenesis mode and then follow selection with extensive characterization, though there have been significant advancements in single-EV characterization technologies.

EV biogenesis kinetics are highly variable; cell type and cell state are primary factors to consider. In a single-cell *in vitro* analysis, some cells secreted little to no EVs, while other cells exhibited “super-secretor” phenotypes and produced ten-times more than an average cell. Furthermore, EV secretion increases proportionally with the number of neighboring cells indicating paracrine signaling effects regulate EV secretion (Ji et al., 2019). *In vitro* live-tracking of transgenic CD63 fused with a pH-sensitive optical (green fluorescent protein) reporter suggests that a single cell can have between 1 and 15 multivesicular endosome-plasma membrane fusion events (intralumenal vesicle release) per minute (~10^3^ to 2 × 10^4^ release events per cell, per day) considering variance within and between cell lines among the three human cell lines tested (Bebelman et al., 2020). Furthermore, the same system showed a change in EV release kinetics by induction of GPCR-dependent histamine signaling (Verweij et al., 2018) indicating that EV release is sensitive to a variety of stimuli. Additionally, *in vitro* tracking of 10^5^ prostate cancer cells over 10^3^ s showed 2.36 × 10^6^ EVs released with an average of 1.4 EVs per cell per minute (Stratton et al., 2014) giving comparable estimates as described by Bebelman and Verweij et al. If we assume that a single fusion event releases 5 EVs, then we can approximate between 5 × 10^3^ and 10^5^ EVs are being produced per cell, per day by the endosomal pathway/CD63+ EVs alone. If we make comparable estimates with an adherent cell culture system that yields approximately 10^10^ EVs per million cells, per day, then we can numerically approximate 10^4^ EVs produced per cell, per day. Considering that these are immortalized, transfected cell lines, they may have a much different EV release rate than a physiologically healthy cell; however, it provides a useful model to approximate EV biogenesis kinetics. It is also important to note that cell surface area, volume, and osmolality values are tightly regulated (Lloyd, 2013; Cadart et al., 2019; Neurohr et al., 2019), and therefore high rates of EV release are not sustainable without an opposing uptake or cellular remodeling process. The simplest physiological solution is to equate cellular EV uptake and release, though we recognize that there are several other possibilities. Mechanistically, cells could in theory sense the sum of cellular uptake, and maintain equilibrium by releasing EVs with a determined size distribution, osmolality, and frequency. Assuming that EV biogenesis operates in a steady-state kinetic fashion, that an average adult human weighing 70 kg contains 3.7 × 10^13^ cells (Bianconi et al., 2013), 20L of extracellular fluids, and circulating extracellular fluids yielding between 10^9^ to 10^12^ EVs per mL, we can consider that there is a steady-state content of between 1 and 2 × 10^3^ EVs attributable to a single cell at any time, and a balanced production and decay rate of ~10^4^ EVs per cell, per day. Furthermore, using the 0.25 pg average mass of a single EV estimated by Stratton et al. (2014) implies that there can be kilograms of EVs in steady-state, and a total mass flux of ~100 kg per day. Empirical studies also support that EVs have a high turnover rate, with an estimated serum half-life of 7 min in mouse models (Matsumoto et al., 2020). The large EV production/decay rate relative to steady state EV concentration indicates highly dynamic instability and temporal resolution of EV-contained information.

Many groups have hypothesized about EV fates once they are released into circulation and there is limited direct evidence to support any hypothesis. Recent studies are examining EV circulation dynamics in the zebrafish *(Danio rerio)* embryo model system enabling *in vivo* live-tracking of single endogenous EVs (Verweij et al., 2019). Verweij et al. were able to observe yolk syncytial layer-derived EVs being produced, entering blood circulation, and being adsorbed to the caudal plexus epithelium where they subsequently underwent either of two fates. In one pathway the EVs were endocytosed by patrolling macrophages. This observation supports the hypothesis that EVs function as long-distance immunological signal carriers (immune surveillance hypothesis). If the RNA contents of EVs are in fact delivered to recipient cells, it is challenging to determine what physiological effect they may have, considering stoichiometric studies which show that any given transcript is present at or less than a frequency of one per EV (Chevillet et al., 2014; Wei et al., 2017; He et al., 2019). In the other case, they observed endothelial cell EV uptake and lysosomal fate (Verweij et al., 2019). This evidence supports that at least some EV subtypes are degraded on delivery and cannot functionally transfer RNA as reported by Kanada et al. (2015), and brings up a number of questions regarding what functions EVs perform and how those functions are accomplished. Furthermore, exogenous EVs originating from the same tissue type had the same trafficking route and fate (Hyenne et al., 2019; Verweij et al., 2019). From these *in vivo* studies, we can infer that EVs have a cell type of origin-specific fate and functional program.

New evidence related to EV transport through the extracellular environment, a matrix with an effective pore size smaller than many EVs, suggests that EVs have unique mechanical interactions within confining matrices which enable their escapement (Lenzini et al., 2020). First, EV escape from a matrix is mechanosensitive. Paradoxically, EVs released more effectively from, and traveled faster through a stress-relaxing hydrogel with a high complex shear modulus (stiff matrix) than a lower one (soft matrix), or one without stress-relaxation properties. In contrast, polystyrene nanoparticles and liposomes exhibited conventional mechanics and moved slower through a stiff matrix than a soft one. Individual EVs in a stiff, stress-relaxing matrix also had a large variance in their diffusion coefficient over time, but an average speed close to nanoparticles moving freely in solution, indicating a dynamic instability in their entrapment. Furthermore, knockdown of the aquaporin AQP1 in EVs significantly impeded particle motion, suggesting that aquaporin-dependent EV deformability is crucial for EV transport (Lenzini et al., 2020). These experiments by Lenzini et al. collectively show that there is still much to study with regard to basic EV transportation.

## 3. Experimental Approaches

**Figure 3:**
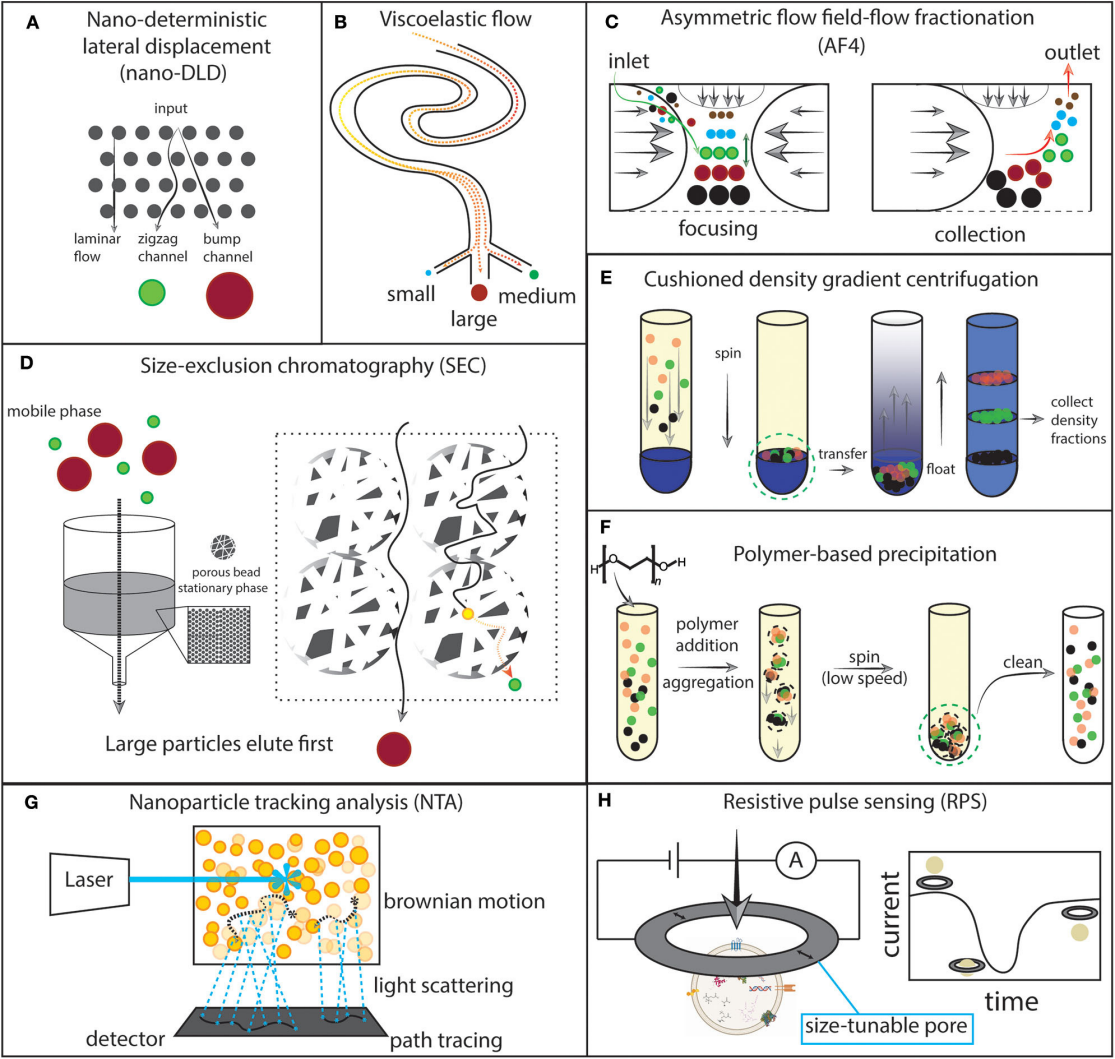
Schematics of selected experimental approaches for extracellular vesicles (EVs). **(A)** Nano-deterministic lateral displacement (nano-DLD). EVs are passed through a regularly-interspaced micropillar array with laminar flow. The pillar size and spacing determines how EVs of a specific size will migrate through the array. Smaller EVs output at the zigzag channel while larger EVs output at the bump channel. **(B)** Viscoelastic flow. Particles flowing through a viscoelastic medium are forced to their equilibrium position in the fluid channel and can then be collected. **(C)** Asymmetric flow field-flow fractionation (AF4). Opposing parabolic flows and an orthogonal flow focus particles to the center of the channel and then particles migrate to an equilibrium position. Then, the opposing parabolic flow is removed and particles elute from small to large. **(D)** Size exclusion chromatography (SEC). A stationary phase is built by packing nanoporous beads into a column. The biofluid is eluted in the mobile phase. Small particles take a longer path through the column by traversing through the beads, while larger particles travel outside of the beads. **(E)** Cushioned density gradient centrifugation. The sample is layered over a high density medium, then spun. Particles collect at the cushion made by the interface of the high density medium and the sample medium. The particles are then transferred to the bottom of a tube and layered with a density gradient. Upon centrifugation, the particles float upward to their equilibrium density position. The density fractions can then be collected. **(F)**. Polymer-based precipitation. Addition of a volume-excluding polymer to the sample induces aggregation and precipitation which then allows for low-speed centrifugation to collect the precipitated particles. The sample can then be cleaned of the volume-excluding polymer and other potential contaminants. **(G)** Nanoparticle tracking analysis (NTA). A laser is shone onto the sample and scattered photons are detected continuously by video. Brownian motion is traced and correlated with particle properties. **(H)** Resistive pulse sensing (RPS). A current is applied to a nanopore and recorded over time. EV motion through the pore results in a measurable current drop which can then be correlated with particle properties.

EV studies contain a vast amount of complexity in their experimental approaches (Figure 3), which has challenged both their validity and the reproducibility of published findings. First, EVs have generated excitement surrounding their biological capabilities in a variety of contexts. Second, the intrinsic difficulties of working with EVs have led to a wide array of technological innovation. In concert, these factors have brought investigators from many different disciplines to enter the field. The diversity among EV studies ultimately is what challenges their validity and reproducibility. For detailed guidelines to both designing EV studies and critically interpreting them, see the Minimal Information for the Studies of EVs (MISEV) 2018 position statement (Théry et al., 2018). We review the key points below.

The first principle of experimental EV work is to identify the appropriate separation and/or concentration method(s) to address the research needs; these methods generally fall into four categories: centrifugation, chemical precipitation, microfluidics, and biochemical capture. MISEV2018 guidelines emphasize the adoption of the terms “separation” and “concentration” when discussing EV experimental methods. A separation technique selectively removes EVs from other fluid components, or one EV subtype from another (e.g., anti-CD63 capture beads). A concentration technique increases the EV concentration in the fluid and may not necessarily remove EVs from other fluid components (e.g., a size-exclusion filter). The MISEV2018 authors established a heuristic to conceptualize the efficacy of different methods by classifying extracellular particle recovery (i.e., concentration) and specificity (i.e., separation) as either low, intermediate, or high efficacy. Each method has some component of separation and concentration regardless of whether the properties are explicitly stated. High-recovery and high-specificity together in a single method is unlikely to be achievable. EV size, density, surface markers, biofluid type, sample volume, product purity, and cost are all to be considered since every method has its own advantages and disadvantages (Théry et al., 2018). The 2015 ISEV survey showed that differential ultracentrifugation was the most popular singular method, being used by 80% of ISEV members surveyed while 60% of respondents also cited using a combination of methods. As expected, cell culture media was the most common starting material and used by the majority of respondents, suggesting that investigators using less frequently studied biofluids should take extra care. Interestingly, Gardiner et al. gathered that of researchers using precipitation techniques, 84% subsequently perform RNA analysis (Gardiner et al., 2016) suggesting that concentrating EVs alone is often acceptable in RNA studies. The MISEV2018 guidelines and the 2015 ISEV survey make salient that separating EVs from the complex milieu of extracellular fluids is a significant problem and in most cases requires a combinatorial approach specific to the research question.

Standardized EV characterization is key for the community to ascribe EVs to experimental outcomes with proof beyond reasonable doubt. As with isolation and concentration methods, EV characterization methods continue to build in complexity as investigators aim to understand EV heterogeneity and biological insights at a deeper level. The MISEV 2018 guidelines for EV characterization follow these four principles:

1. Quantitatively describe the EV source (e.g., cell count, fluid volume, method employed) and product yield (e.g., macromolecule quantities, particle count).
2. Generally characterize that the sample contains vesicles and describe the purity of the preparation. Vesicle existence can be claimed by demonstrating that the sample contains intact lipid bilayers, that are unlikely the products of cell fragmentation. For example, the identification of at least one membrane-associated protein (e.g., TSG101) and a transmembrane (e.g., CD63) or lipid-anchored (e.g., sonic hedgehog) protein would be sufficient to meet this criterion. Investigate the presence and extent of non-EV contaminants e.g., lipoproteins (targets depend on EV source and preparation method).
3. Employ a combination of single EV characterization techniques. Ideally use an optical (e.g., electron microscopy, nanoparticle tracking) and a biophysical/biochemical (e.g., resistive pulse sensing, Raman spectroscopy) modality tailored to the EV product and considering the limitations and complementarities of each technique.
4. When considering EV-associated biological functions, it is necessary to operate with the utmost detail and rigorous experimental design. Functional studies should be performed with strict biofluid fractions; select the appropriate method(s) to separate EVs and EV subtypes from non-EV contaminants. Test the purported activity with condition-matched controls (e.g., healthy vs. disease-derived sample) of the biofluid itself, the EV-depleted biofluid, crude EVs alone, and EV subpopulations if relevant. Evaluate the relevant molecular topologies (i.e., externalized or internalized molecular functionality) for membrane-bound molecules.

A strategic approach to EV characterization is essential to advancing the field (Théry et al., 2018).

### 3.1. Separation and Concentration Methods

EV separation and concentration is the most essential step to working with EVs. Each method imposes some types and degrees of bias on what kinds of EVs and other contaminants will be present in a sample, and these biases are carried through the rest of a given study (Van Deun et al., 2017; Brennan et al., 2020). Studies using modern approaches to EV separation and concentration, which are typically combinatorial in nature, are illuminating the limitations of prior single-method studies (Jeppesen et al., 2019). The advantage of combining several techniques is that some extracellular particles have a size overlap with EVs, while others have a density overlap, and typically a method will select for either a specific size or density, but not both. To study an EV sample of the highest purity, it is necessary to employ at least two techniques that together can select for a specific size and density (e.g., a size-exclusion filtration followed by density gradient centrifugation, Figure 3E). Furthermore, the translation of EV research into clinical laboratories depends on creating a streamlined and highly-reproducible process for preparing EV samples. In our literature review, we have not found any apparatus which can take a whole biofluid as an input, and output high-quality EV preparations in a single step. The methods reviewed below either rely on sample pre-processing or multiple step processes to make EV preparations. Therefore, there is significant opportunity for innovating a device or process to prepare EVs in a single step and streamline clinical implementations. In this section, we review the newest and most common methods for EV separation and concentration.

#### 3.1.1. Differential Ultracentrifugation

Differential ultracentrifugation (DUC) is a mainstay EV separation technique. The operating principle of DUC is to apply step-wise increases in centrifugal force to a solution. The product of centrifugal force and time during each step then concentrates particles of a specific size and buoyancy. Ideally, using this principle, one can separate particles within a solution into discrete fractions. For example, a cell fraction, a large vesicle fraction, and a small vesicle fraction. Since DUC is a relatively simple and pure technique, it is the established EV isolation standard. The primary benefit of DUC is the ability to concentrate and separate EVs by simply subjecting the biofluid to high rotational speeds. Furthermore, DUC parameters such as rotor type, rotational force, solution density, and spin time can be finely adjusted to meet the needs of a specific application, for example a specific biofluid type and EV fraction. Most DUC protocols include some variation of a three-step method including a low-speed ~1,000 RCF spin to collect cells and other large particles, an intermediate-speed ~20,000 RCF spin to collect large EVs, and a high-speed ~100,000 RCF spin to collect small EVs. The most notable innovation/adoption among centrifugation-based protocols is iodixanol-based density gradient ultracentrifugation (DGUC) (Figure 3E) which has allowed researchers to select EVs of a specific density with higher precision than could be achieved with classical DUC, even with derivative techniques such as sucrose gradients or cesium chloride gradients (Li et al., 2018b). Iodixanol DGUC studies have been critical in elucidating EV heterogenity. An early study using iodixanol DGUC identified a number of purported small EV biomarkers which were also found in large EVs (Kowal et al., 2016). Newer evidence has shown that 10 of the top 25 proteins associated with EVs were actually detected in non-vesicular nanoparticles and not in EV fractions (Jeppesen et al., 2019). To this end, iodixanol DGUC studies highlight the continued strength of centrifugation-based protocols and the importance of method selection when seeking to separate and characterize EV subtypes.

Centrifugation-based protocols face a number of limitations that have driven innovation to create new methods:

1. The amount of variance and lack of standardized protocols within the field presents a challenge for validating findings made between groups and a barrier to entry for new groups. Commercially available isolation kits (using a variety of methods, e.g., precipitation, chromatography, etc.) are one solution aimed at the reproducibility problem.
2. DUC protocols for EV isolation are lengthy, taking at least several hours.
3. Only a small fraction of EVs are captured by DUC, which can make investigations of rare samples difficult (Liu F. et al., 2017). Below, we review several methods that have focused on optimizing processing time and yield.
4. Standard DUC is insufficient for high-resolution and high-fidelity EV separation studies in complex biofluids due to EVs overlapping in size and density with other biological nanoparticles (Jeppesen et al., 2019). Preparing EV samples of high purity generally requires either a combination of methods or more complex derivatives of existing methods (Théry et al., 2018).
5. DUC subjects EVs to extremely high forces (typically 1 − 2 × 10^5^ RCF) which many people suggest may irreversibly damage EVs or change EV properties studied thereafter (Li et al., 2018b). We review a number of techniques which do not require high forces (e.g., precipitation, chromatography, etc.) or protect the EVs (e.g., cushioned centrifugation, Figure 3E) from high forces.
6. The capital cost to acquire an ultracentrifuge can prevent its widespread adoption in clinical and research laboratories. Commercially-available kits are another solution that reduce or eliminate the large capital investment required for EV-based assays.

Despite all of these drawbacks, centrifugation continues to be the reference standard for innovating methods.

#### 3.1.2. Precipitation

EV precipitation strategies are advantageous over ultracentrifugation because they often recover a larger fraction of EVs, and they can function with standard benchtop centrifuges. Reports describing chemical precipitation of virions and other nanoparticles by volume-excluding polymers e.g., polyethylene glycol (PEG) date as early as the 1970s. Alice Adams reported that the Epstein-Barr virus could be recovered with high yield and retained viral activity when separated directly from cell culture medium using PEG-based precipitation, while sucrose density gradient centrifugation had low recovery and substantially attenuated viral activity (Adams, 1973). The operating principle of polymer precipitation is that introduction of the polymer decreases the volume of solvent available to other particles, thereby increasing the native particles' effective concentrations until they precipitate from the solution (Mahadevan and Hall, 1992) (Figure 3F). Several companies including System Biosciences (Antes and Kwei, 2012) and ThermoFisher Scientific (Vlassov et al., 2014) have adapted the PEG precipitation method to efficiently cluster and precipitate EVs from specific biofluids. The protocols use only low-speed (<10^4^ RCF) centrifugation and take about 1 h to complete EV isolation from blood plasma with newer kits. PEG-based precipitation methods have been shown to yield hundreds of times more EVs than centrifugation and therefore can be used effectively in low sample input applications (e.g., 250 μL of plasma; one drop is about 60 μL)^2^. Accordingly, polymer precipitation has been widely adopted over the past 10 years because of its relative ease. However, the principal problems faced by polymer precipitation are the formation of EV and non-EV aggregates which then co-precipitate with EVs, and solution contamination by the polymer which can skew downstream analyses (Brown and Yin, 2017). While polymer introduction contaminates the sample to some extent, using a higher average molecular weight polymer tends to yield cleaner final products (Mahadevan and Hall, 1992). Furthermore, the precipitate can be cleaned using column-based chromatography which means that contamination may not be a precluding factor for the widespread adoption of this method. Though we will not review them here, examples of non-volume-excluding polymer precipitation techniques include the Vn96 polypeptide (ME kit, New England Peptide) which binds EV-associated heat-shock proteins causing aggregation (Griffiths and Lewis, 2015), and charge-based precipitation using protamine (Deregibus et al., 2016). Overall, precipitation strategies have great potential in low sample input scenarios and where EV purity is not of the highest priority.

#### 3.1.3. Biochemical Capture

Biochemical capture techniques are most appropriate for investigators who seek to subset EV populations by specific membrane components and ascribe functions to those EV sub-populations (e.g., CD63-high EVs can elicit some biological response while CD63-low EVs cannot). Biochemical capturing of EVs in most embodiments depends on immunoaffinity capture (IAC), where antibody-binding interactions dictate which particles become concentrated. The IAC approach uses antibodies conjugated either with the surface that the fluid sample passes over (Reátegui et al., 2018), or to beads that mix with the sample and then are collected (Shao et al., 2012). IAC surfaces efficiently separated EVs from plasma (Reátegui et al., 2018), but the limitation of IAC surface designs to EV isolation largely prevents their popularity. IAC beads implemented strategically can both separate and label EVs for downstream analysis, therefore IAC beads are often favored over IAC surfaces (Shao et al., 2012; Oksvold et al., 2015). Dynabeads are a popular polymer-based bead that can be synthesized with high fidelity in a desired size range, with specific chemical properties such as superparamagnetism, and conjugated with antibodies or antibody linkers to be used for IAC. For more detail on Dynabeads and bead-based EV capture (see Ugelstad et al., 1987; Jorgedal et al., 2007; Oksvold et al., 2015). Due to the extensive EV heterogeneity we are beginning to uncover with single-EV analytics (Lee et al., 2018; Ji et al., 2019), both the strengths and weaknesses of biochemical specificity are becoming apparent. We suspect that IAC and biochemical capturing techniques as a whole are limited with regard to biological insights, insofar as their outcomes may not be generalizable between biological systems.

#### 3.1.4. Microfluidic Technologies

Microfluidic (MF) technologies use physical properties to manipulate solutions on a small (μL-pL) scale^3^. MF-based EV separation and concentration technologies currently exist in a number of embodiments including filtration, chromatography, nanowire trapping, nano-deterministic lateral displacement, viscoelastic flow, acoustic separation, and asymmetric flow field-flow fractionation (AF4). This section describes the principles of each approach listed, examples of their implementation within EV research, and their limitations.

##### 3.1.4.1. Filtration

The most simple manifestation of MF technology applied to EV separation/concentration is filtration. In this method, a solution is forced through a nanoporous filter material where particles larger than the filter's effective pore size are concentrated on the filter, while smaller particles pass through and remain in the filtrate solution. Particles of a specific size range can be separated from particles outside of that range by implementing a filter series where each filter has different effective pore sizes. Many groups use filtration alone or in combination with other techniques such as centrifugation or precipitation for greater power to select specific EV subsets. A device with low protein binding, track-etched polycarbonate membrane (Apel, 2001) filters, was shown to separate EVs with high fidelity and efficiency from human clinical samples including plasma, lavage, and urine (Liu F. et al., 2017). Another group demonstrated EV isolation from whole blood by electrophoresis-driven filtration which enhanced RNA extraction per unit of protein (Davies et al., 2012). Filtration approaches are popular because of their simplicity but challenging due to filter clogging and vesicle deformability under pressure.

##### 3.1.4.2. Chromatography

Chromatographic techniques, which can be further divided into size-exclusion and affinity modes, are widely employed for EV separation protocols and have a marked advantage over other methods we review here because they can yield very clean and highly-reproducible products. In traditional size-exclusion chromatography (SEC), a solution (the mobile phase) travels through a column packed with porous resin beads (the stationary phase). Small particles can enter the resin pores, and thus have a longer path to elution, while larger particles travel a more direct path outside of the beads and elute more quickly (Figure 3D). For a practical primer on SEC (see Burgess, 2018). SEC of platelet-depleted plasma using a hand-packed Sepharose CL-2B column (fractionation range: 100 kDa–20 MDa) was capable of enriching platelet-derived EVs vs. plasma proteins by hundreds of times, and vs. HDL-cholesterol by a factor of about ten times (Böing et al., 2014). However, SEC preparations still require additional treatment to ensure substantial elimination of lipoproteins (Karimi et al., 2018). SEC methods function with limitations similar to filtration methods, namely that the pore size is deterministic and therefore selects EVs (and other particles) of some sizes preferentially over others. In the case of Izon Science's qEV column, a 35 nm effective pore size recovers more small EVs (< 110 nm) with the trade-off of greater potential contamination by lipoproteins. Conversely, a 70 nm effective pore size depletes both small EVs and lipoproteins while enriching larger EVs (> 110 nm). Overall, we find that SEC columns are a relatively high-fidelity technique for separating EVs from other biofluid components.

In affinity chromatography, the mobile phase travels through a stationary matrix exhibiting an affinity for certain particle types; the interaction can be general (e.g., negatively charged particles) or specific (e.g., antibody-epitope binding). High affinity particles are retained non-covalently in the stationary phase upon sample application. Then, graduated elution of a solution with comparable affinity displaces adsorbed particles once the bonding interaction is overcome. Zeta-potential measurements of EVs by both resistive pulse sensing and electrophoretic mobility assays have indicated that EVs carry a negative surface charge (Kozak et al., 2012; Deregibus et al., 2016; Vogel et al., 2017; Jamaludin et al., 2019), informing the development of affinity columns that leverage electrostatic interactions for capturing EVs. We discuss zeta-potential further in section 4. Patents related to the QIAGEN exoRNeasy kit describe a regenerated cellulose membrane functionalized with quaternary ammonium cations (Enderle et al., 2015a). The QIAGEN membrane affinity column yielded a similar number of EVs with less non-vesicular protein contamination than DUC-prepared EVs (Enderle et al., 2015b). Affinity columns have an advantage of biochemical flexibility over SEC columns: stationary phase materials have extensive flexibility with regard to their composition, and functionalization by antibodies, aptamers, and other moieties (Block et al., 2009; Urh et al., 2009; Acquah et al., 2015; Tanaka and McCalley, 2016). Low-input chromatographic systems have also been demonstrated and we expect to see future development in microfluidic, chromatography-based modules with further specialization for handling clinical samples and extracting RNA (Chirica et al., 2006; Millet et al., 2015; Surawathanawises et al., 2017). Overall we find that chromatographic EV separations, especially those prepared using commercial kits, have a high applicability to discovery- and EV-based RNA studies where purity and reproducibility are paramount.

##### 3.1.4.3. Nanowire trapping

Nanowire trapping has been tested in proof-of-concept studies for EV separation and concentration, but we have yet to see its adoption in RNA-sequencing (RNA-seq) studies. The principle is to construct an obstacle network which selectively traps EVs while allowing undesired fractions to pass through. Then, the network integrity is dissolved or perturbed to release the trapped EVs. An early embodiment used ciliated silicon micropillars in a regularly-interspaced (0.9 μm) array with a tunable capturing range of 30–200 nm. The nanowire forest captured EV-like liposomes, while other cellular and protein debris flowed through. Then, the liposomes were released by incubating the MF channel with phosphate-buffered saline, thereby dissolving the silicon nanowires. This architecture was additionally flexible in that antibodies could be loaded on the nanowires; however, it was not demonstrated (Wang et al., 2013). Furthermore, the 24-h period required to significantly dissociate the silicon nanowires for EV collection is a considerable challenge with regard to competing with other techniques. A recently-described polypyrrole nanowire architecture was able to overcome this kinetic limitation (Lim et al., 2019). In the polypyrrole embodiment, the wires were divided into apical and basal domains linked with circulating tumor cell-related and EV-related antibodies respectively. Small EVs (<100 nm) were trapped while larger EVs were excluded. The polypyrrole chemistry was such that disulfide bridge reduction by 50 mM glutathione treatment for 30 min released the circulating tumor cells, while electrical stimulation at −1.5 volts for 3 min released the EVs. The device was capable of yielding 4 × 10^9^ EVs per mL, meeting or exceeding the performance of commercial EV precipitation kits (Lim et al., 2019). Nanowire trapping has a demonstrably flexible design capability as evidenced by these studies insofar as it can select for EVs by both size and biochemical characteristics.

##### 3.1.4.4. Nano-deterministic lateral displacement (nano-DLD)

Nano-deterministic lateral displacement (nano-DLD) is a promising technology which has already been used to concentrate EVs within human biofluids for RNA-seq analysis (Murillo et al., 2019). In nano-DLD, laminar flow drives a solution through a regularly-interspaced micropillar array. Dissolved particles follow a deterministic, size-dependent path through the array and become concentrated in the collecting outlets (Huang et al., 2004) (Figure 3A). EV sorting by nano-DLD was conceptualized with a 235 nm micropillar spacing architecture in a proof-of-concept study that primarily relied on polystyrene beads (Wunsch et al., 2016). The 2016 design had limited results with EVs and a throughput limit of 0.2 μL per hour at 10 bars of operating pressure making it impractical for biomedical applications (Smith et al., 2018). A follow-up study scaled the design to include 1,024 nano-DLD arrays on a single chip, allowing a throughput rate of 900 μL per hour with 225 nm spacing and 10 bars of operating pressure. Furthermore, the device was tested with whole biofluids. The nano-DLD chip was competitive with centrifugation and chromatographic techniques, concentrating EVs from serum and urine by a factor of three with ~70% yield (Smith et al., 2018). RNA-seq analysis of serum from human prostate cancer patients indicated that RNA preparations by nano-DLD had higher reproducibility than those which were prepared by DUC suggesting that nano-DLD is a potentially scalable solution for research settings and liquid biopsy applications. A third iteration demonstrated the flexibility of the nano-DLD platform by incorporated 3,084 nano-DLD arrays with a smaller bump channel to increase the concentration ability. The new design operated with lower throughput (26 μL per minute) but concentrated EVs 60-fold from urine demonstrating that nano-DLD architectures can be optimized for specific biofluid qualities and desired outcomes (Smith et al., 2018). The limitations of nano-DLD include relatively low sample throughput and susceptibility to blockage due to small (~200 nm) channel sizes (Liu C. et al., 2017), however, scalable manufacturing as described (Smith et al., 2018) and biofluid pre-filtration (Murillo et al., 2019) may address these concerns. We expect to see wider adoption of nano-DLD chip-based EV concentration protocols in future RNA-seq studies.

##### 3.1.4.5. Asymmetric flow field-flow fractionation (AF4)

Asymmetric flow field-flow fractionation (AF4) has great potential for separating EVs from other nanoparticles though its implementation in EV research has thus far been limited. Field flow fractionation first was described in 1966 (Giddings, 1966) and has developed into a number of derivative techniques including AF4. The strength of AF4 lies in its ability to separate particles over a wide dynamic range (Fraunhofer and Winter, 2004). The operating principle is to focus particles by subjecting them to opposing, parabolic channel flows, plus a perpendicular “cross-flow” which then elutes through a semi-permeable membrane (Zhang and Lyden, 2019) (Figure 3C). Most notably, AF4 has resulted in the identification of exomeres, non-vesicular extracellular nanoparticles which co-isolate with EVs in most instances and have demonstrated biological activity (Zhang H. et al., 2018; Zhang and Lyden, 2019; Zhang Q. et al., 2019). Despite the specialized nature of AF4 protocols (Zhang and Lyden, 2019), we expect to see wider adoption of AF4 in EV-based RNA studies because of its ability to handle very small particles which are often lost with other techniques.

##### 3.1.4.6. Viscoelastic flow

The strength and flexibility of microfluidic devices that leverage viscoelastic fluid properties have been demonstrated in recent years for nanoparticle sorting (Kang et al., 2013; Lim et al., 2014; Liu et al., 2016; Zhou et al., 2020), and specifically for separating EVs (Liu C. et al., 2017; Zhou Y. et al., 2019; Asghari et al., 2020). Viscoelastic focusing is a phenomenon where the flow of a dilute polymer solution, which carries both elastic and viscid properties, can generate forces including elastic lift that then push particles of a specific size and rigidity to an equilibrium position in the fluid channel (Leshansky et al., 2007) (Figure 3B). In one instance, EV separation by viscoelastic flow was performed on-chip by addition of 0.1% PEG (600 KDa average molecular weight). The viscoelastic separator exhibited high recovery of EVs from native fetal bovine serum, demonstrating its ability to function with minimally processed biofluids (Liu C. et al., 2017). Recently, Zhou et al. presented a wavy microfluidic channel geometry which added secondary lateral forces and thereby focused larger particles three times more than previous designs (Zhou Y. et al., 2019). Furthermore, by controlling the fluid flow rate and PEG concentration with the same microfluidic channel geometry, they demonstrated a tunable device that can select cells and particles of specific sizes from a mixture (Zhou et al., 2020). In the future, we anticipate that this concept will be tested on EVs.

Most viscoelastic strategies are challenged to focus and separate < 100 nm particles due to increased diffusivity and decreased elastic forces. A sheathless, oscillatory design was shown to focus 20 and 40 nm particles, though it was not shown that they could be separated. This device could distinctly separate small EVs (mean diameter = 122 nm) from large EVs (1–2 μm milk fat globules) (Asghari et al., 2020). The flexibility of viscoelastic focusing can also be seen in diversity of viscoelastic fluid preparations. Kang et al. demonstrated particle separation in solution comprised of 0.0005% (w/v) lambda DNA (Kang et al., 2013). Viscoelastic focusing has a distinct advantage over other kinds of focusing methods insofar as it functions passively, without applying an external field. Therefore, viscoelastic separators can be highly portable and easily manufactured (Liu C. et al., 2017; Zhou Y. et al., 2019; Zhou et al., 2020). We see great potential in the usage of viscoelastic microfluidic devices for future studies of EV-derived RNA from clinical samples, given their natural ability to work with biological fluids.

##### 3.1.4.7. Acoustic separation

Acoustic field-based separation and concentration of EVs has been proposed in a number of realizations (Wu et al., 2017; Habibi and Neild, 2019). Conventionally, acoustic isolation technologies subject the fluid to differential acoustic forces and particles are laterally displaced in proportion with their size. Recently, a sound wave activated nano-sieve was estimated to have 50-fold nanoparticle enrichment capability (Habibi and Neild, 2019). The advantages of acoustic isolation include straightforward manufacturing, and contactless particle separation (Wu et al., 2017). Given advancements in acoustic tweezer technologies (Lutz et al., 2006; Ozcelik et al., 2018; Zhang S.P. et al., 2018) we expect to see future innovation in this area.

### 3.2. Characterization Methods

As described above, a strategic and standardized approach to EV characterization is essential to advancements in the field–specifically for the translation of EV research into biomedical innovations. In this section, we provide a brief overview of analytical techniques that characterize EVs physically and biochemically. For additional perspectives on technologies and approaches for EV analysis, see the following review and the MISEV2018 guidelines (Shao et al., 2018; Théry et al., 2018).

#### 3.2.1. Physical Characterization

Nanoparticle tracking analysis (NTA) is now a mainstay for EV counting and sizing that is based on visible light microscopy. NTA devices shine a laser through the sample and record a video. Brownian motion of individual EVs, which can be inferred by the way light scatters through the sample over time, directly relates to their particle size (Figure 3G). Therefore, NTA can estimate both the particle concentration and size distribution within a sample based on direct observation of particle motion (Dragovic et al., 2011). In comparing two of the NTA market leaders, Particle Metrix's ZetaView and Malvern's NanoSight, Bachurski et al. found that the ZetaView was more reliable for EV concentrations while the NanoSight reported more accurate particle sizes (Bachurski et al., 2019). Newer NTA devices are capable of measuring particle motion under an applied electric field, which allows calculation of the zeta-potential, a proxy for particle surface charge^4^. Interestingly, Bachurski et al. found that NTA consistently overestimated EV sizes vs. TEM even when accounting for 11–20% volume loss during TEM sample preparation (Doughty et al., 1997; Bachurski et al., 2019). The analysis suggests that NTA should not be considered an absolute reference for EV sizing. Furthermore, NTA studies have reported a practical detection limit of 60–70 nm (van der Pol et al., 2014; Bachurski et al., 2019), while TEM analysis found the mode particle size of serum-derived EVs to be 50 nm (Bachurski et al., 2019), indicating that smaller particles are either being lost or miscounted during light-based EV characterization. Given recent studies raising the importance of particles <50 nm (Jeppesen et al., 2019; Zhang Q. et al., 2019), we can significantly benefit from high-throughput methods, optical or otherwise, which can accurately size particles over a wide dynamic range including <50 nm.

There are a number of newer optics-based methods for EV characterization. Similar to NTA, single particle interferometric reflectance imaging (SP-IRIS) functions by capturing EVs on an antibody microarray chip. Light is shone onto the chip and then the reflectance is measured. Interference between the reference light-field, produced by reflectance off of the silicon chip alone, and the scattered light field produced by reflectance off of the EV-bound microarray, gives a signal which corresponds to the EV size at each location on the chip (Avci et al., 2015; Daaboul et al., 2016). While SP-IRIS may produce more reliable measurements than NTA (Bachurski et al., 2019), the distinct advantage of NTA over SP-IRIS is its label-free function. Separation-free EV quantification is another area which is important to consider, since current separation methods each favor certain EV subsets over others. Dye-labeled aptamers with affinity for CD63 accurately quantified EV concentrations between 5 × 10^2^ and 5 × 10^5^ per μL based on the change in fluorescence polarization (Zhang Z. et al., 2019). The polarization assay is advantageous insofar as it can effectively quantify EVs in whole human plasma; however, it still depends on specific marker labeling. There has been considerable effort to apply flow cytometric technologies toward EV characterization. While most nanoscale and imaging flow cytometry methods operate with a lower limit of detection at 100 nm, there are reports of a custom-made flow cytometer with a 40 nm detection limit (Zhu et al., 2014; Ma et al., 2016; Tian et al., 2018). Flow cytometric techniques are beginning to elucidate single-EV biology (Marcoux et al., 2016; Stoner et al., 2016; Saugstad et al., 2017; Mastoridis et al., 2018; Tian et al., 2018; Kanada et al., 2019; Padda et al., 2019; Ricklefs et al., 2019; Zaborowski et al., 2019) and we expect to see significant growth in this area. There have also been a number of notable studies implementing nanoplasmonic technologies for single-EV detection and characterization (Im et al., 2014; Raghu et al., 2018; Rojalin et al., 2019).

Resistive pulse sensing (RPS) is another commonly implemented technique for EV counting, sizing, and surface charge characterization. RPS principally operates by electrophoretic translocation of particles through a conical pore; when within the pore, the particle increases electrical resistance across the pore and a current drop is detectable. The magnitude and frequency of current drops are proportional to particle volume and concentration, respectively (Lan et al., 2011) (Figure 3H). Since the description of RPS as a means for counting particles in 1949 (Coulter, 1949), a number of significant innovations have been made to allow for particle characterization on the nanometer scale, namely: microfluidics, manufacturing techniques, and new materials that led to small and size-tunable pores (DeBlois and Bean, 1970; Deamer, 1997; Saleh and Sohn, 2003; Fraikin et al., 2011; Lan et al., 2011; Kozak et al., 2012). Izon Science is the market leader of RPS devices for EV analysis and their device has been tested in a number of peer-reviewed publications. RPS distinguishes itself from other characterization techniques because it measures EVs individually rather than in aggregate. The current device operates in the 40 nm to 10 μm size range^5^. Particle aggregation and pore clogging are some primary limitations of the technology (DeBlois and Bean, 1970; van der Pol et al., 2014). In summary, RPS is a valuable technique for EV characterization since it simultaneously counts and measures the size and zeta-potential of each particle individually (Kozak et al., 2012; Vogel et al., 2017).

#### 3.2.2. Biochemical Characterization

Mass spectrometry (MS), nucleic acid sequencing (NA-Seq), and antibody affinity-labeling techniques are all currently being implemented for high-throughput, multiplexed profiling of EV biochemistry. The advantages of EV-focused MS analysis have been made clear through a number of studies including the identification of GPC1 as a marker of pancreatic cancer-derived EVs, genetic renal disease markers in urinary EVs, and novel myokines secreted during exercise in mammals (Gonzales et al., 2009; Melo et al., 2015; Whitham et al., 2018). Namely, EV separation and concentration prior to MS improves biomolecule discovery and identification by depleting highly abundant proteins and thereby sidestepping the dynamic range problem inherent within MS (Whitham and Febbraio, 2019). By separating EVs into density gradient fractions and then performing MS, for instance, one group was able to more sensitively uncover the heterogeneity among the protein content of EV subsets (Kowal et al., 2016). MS analyses have also been critical for comprehensively examining EV lipid components. Lipids are especially important to consider in small EVs since the membrane thickness is ~5 nm and therefore can take up a significant fraction of the total EV volume (Kreimer et al., 2015). In the next section, we will expound on a number of MS-based discoveries related to EV composition.

NA-seq has been and will continue to be a crucial technology for EV characterization. Next-generation, NA-seq technologies are sensitive to a very wide dynamic range of transcripts (McCombie et al., 2019). Furthermore, because of biochemical techniques such as rRNA depletion, sequence reads become less saturated by rRNA and magnify the detection of other, less abundant RNA species of experimental interest (Wei et al., 2017). EV-derived RNAs contain extensive diversity that is only beginning to be appreciated with the application of NA-seq (Freedman et al., 2016; Wei et al., 2017; Godoy et al., 2018; Vagner et al., 2018; McCombie et al., 2019), and we will discuss these RNA contents further below. The benefits of studying total extracellular RNA profiles are clear: EVs are only one RNA carrier type of many, and accordingly they only show us one aspect of the molecular picture. Yet, taking a reductionist approach to studying each type of RNA carrier, e.g., EVs, also translates to creating essential frameworks and toolkits for understanding the whole system from bottom-up and top-down views, such as we see with the XDec deconvolution platform described by Murillo et al. (2019). Therefore, NA-seq studies with careful attention to EV preparation and purity will continue to make valuable contributions to our understanding of EV biology in the context of RNA transportation.

Targeted studies leveraging antibody-based techniques are also beginning to elucidate single-EV and single-cell derived EV characteristics with high throughput. One group biotinylated EVs and then fixed them to a neutravidin-coated microfluidic chamber for staining. They then applied three fluorescently-labeled antibodies per imaging cycle, and up to 11 protein markers in total per EV (Lee et al., 2018). For single-cell derived EV analysis, a microwell platform with fluorescent, multiplexed antibody barcoding was recently described (Ji et al., 2019). Furthermore, a custom, high-sensitivity flow cytometer was capable of detecting single phycoerythrin-conjugated antibodies, and two-color fluorescence flow cytometry was implemented to analyze EV surface protein expression in plasma-derived EVs from colorectal cancer patients (Tian et al., 2018). These studies are only a few representative examples of innovations we have seen regarding the antibody-based probing and characterization of EVs. In total, MS, NA-seq, and antibody-based techniques are all increasing the scope and resolution with which we can understand EV biochemistry.

### 3.3. Integrated Systems on a Chip for Point of Care Clinical Diagnostics

Medicine is rooted in evaluating a disease state, determining underlying causes, and targeting those causes; however, accurate and precise disease diagnoses are difficult to achieve. Therefore, therapies which target underlying causes may not be properly identified or available. For example, ovarian cancer is classified primarily by histological evaluation of surgically-resected tissue with limited or nonexistent molecular detail. Furthermore, nearly all ovarian cancer cases are treated by surgical resection and/or with a platinum-based chemotherapeutic agent regardless of the underlying tumor biology^6^. Yet in a Cancer Genome Atlas study of only 489 high-grade serous ovarian cancer cases, there were four transcriptional subtypes, three micro-RNA subtypes, and four promoter methylation subtypes identified which pointed to 22 precise treatment options (Bell et al., 2011). It follows that a technology improving access to high-resolution diagnostics will heighten our standard of medicine. As discussed throughout this review, EVs have demonstrated significant potential as a clinical tool if they can be assayed in a streamlined and standardized fashion. Small-scale biofluid manipulation and control using MF system-on-chip (MF-SoC) devices is logical for EV isolation and analysis in clinical settings due to their “sample-in, answer-out” (Chiriacò et al., 2018) capability and low volume requirement. In the future, MF-SoCs can simplify, standardize and integrate the process of EV isolation and analysis into a single handheld device which can process human biofluids, concentrate and purify EVs, and perform biomarker analysis (Chiriacò et al., 2018; Guo et al., 2018). Here, we review devices which have demonstrated sequential EV separation and analysis with relevance to clinical diagnoses.

Investigators affiliated with the Massachusetts General Hospital have published a series of MF-SoC devices which they applied to glioblastoma diagnosis and monitoring. In 2012, Weissleder and Lee et al. reported an MF-SoC which used an IAC approach that enriched glioblastoma-derived EVs and subsequently performed protein profiling by NMR. A crude EV preparation was injected with trans-cyclooctene-conjugated antibodies into the device and washed; then, magnetic nanoparticles (MNPs) conjugated with 1,2,4,5-tetrazine were added, the antibody-bead linkage was achieved by spontaneous cycloaddition, and the EV-antibody-MNP product was formed. The sample was finally washed and analyzed for protein content by nuclear magnetic resonance using a microcoil on the device. The NMR output corresponded to EV protein concentration and type. Proteins were detected with orders of magnitude higher sensitivity than traditional approaches, including flow cytometry, ELISA, and nanoparticle tracking analysis. Additionally, the NMR signature of four EV-associated proteins sensitively and specifically identified glioblastoma vs. healthy patients (Shao et al., 2012). In 2013, Weissleder and Lee's group demonstrated that an MF-SoC platform could separate EVs from packed red blood cell samples, and by detecting the EV concentration using a similar NMR strategy, it could determine the effective age of the blood sample (Rho et al., 2013). The multiplexing capability of this device was unclear and can likely be improved with the multichannel, digital NMR sensor that was recently reported by Weissleder and Lee et al. (Huber et al., 2019). We are interested to see an MF-SoC which can integrate a miniaturized mass spec system and thereby have an ability to detect a broader range of proteins and small molecules (Szyszka et al., 2017). In 2015, Weissleder and Lee's group solidified the value of MF-SoC devices for clinical EV analysis by designing a device which could assay for nucleic acid content. The device performed quantitative analysis of EV-derived mRNA by qPCR (Shao et al., 2015). Glioblastoma-derived EVs were enriched on-chip by antibody-conjugated MNPs in a similar process as described above. EVs were then lysed and RNAs were purified with a silica bead filter. RT-qPCR was performed using on-chip integrated hardware. The longitudinal mRNA expression profiles MGMT and APNG in serum EVs were correlated with responsiveness to the glioblastoma treatment temozolomide, confirming that EVs are useful tools for disease diagnosis and real-time monitoring of treatment efficacy (Shao et al., 2015). Given recent advancements in microfluidic technologies for single cell RNA-seq, we expect to see some translation of this technology to EV analysis in an MF-SoC platform which could both perform massively-parallel transcriptional profiling and RNA velocity determination (Macosko et al., 2015; Zheng et al., 2017; La Manno et al., 2018). In summary, MF-SoCs have demonstrated significant potential for both preparation and analysis of EVs in a clinical setting.

## 4. Extracellular Vesicle Composition

**Figure 4:**
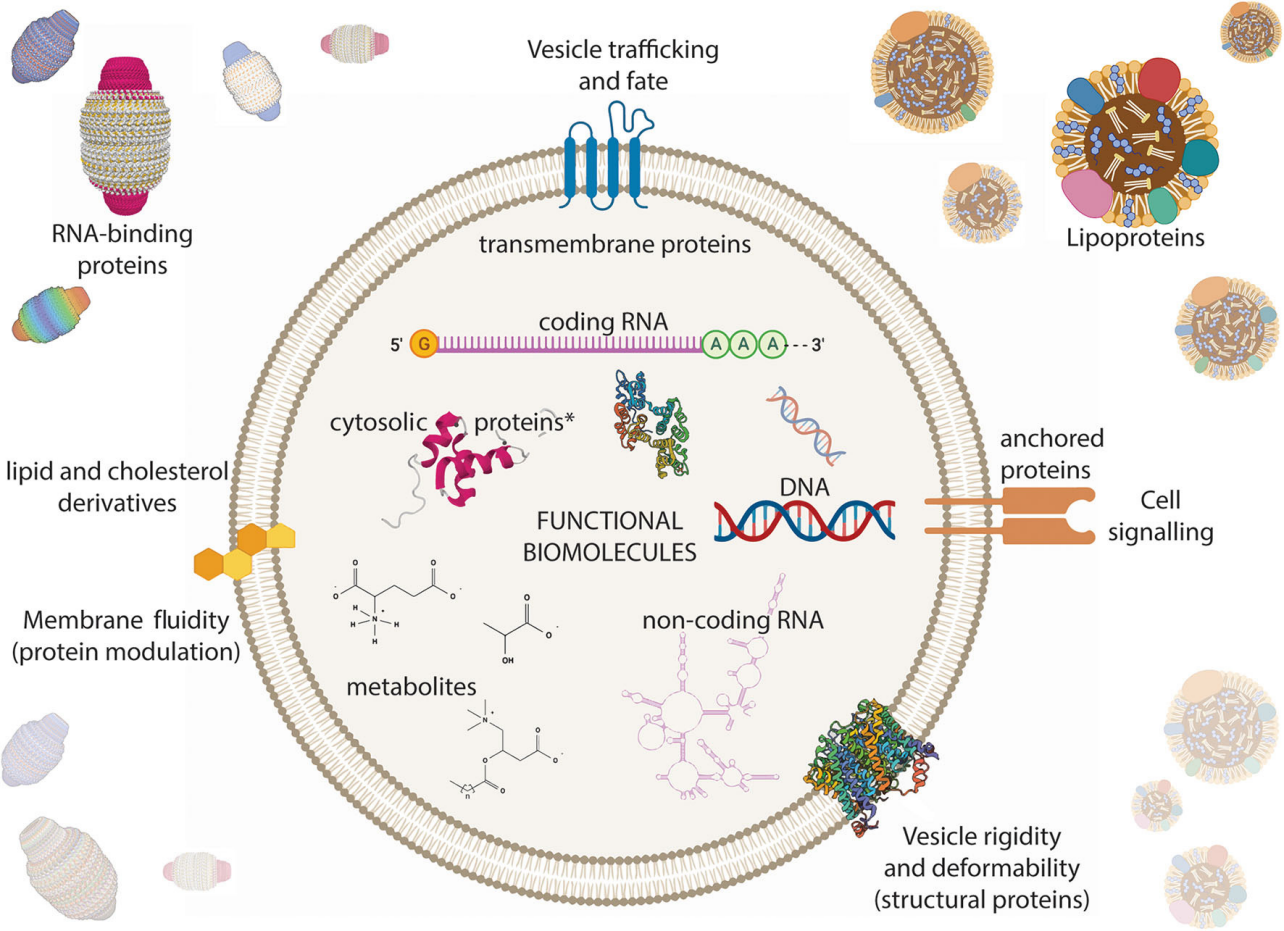
Extracellular vesicle (EV) composition in the context of biological solutions. EVs carry all biomolecule classes that have been associated with cells. DNA and RNAs (both coding and non-coding) are found within EVs. Proteins can be freely soluble, membrane-associated, membrane-anchored, and trans-membrane. Metabolites and other small molecules are also found within EVs. The membrane bilayer is composed of phospholipid and cholesterol derivatives. EVs cannot be purely isolated and other non-vesicular molecular chaperones such as RNA-binding proteins and lipoproteins often contaminate EV preparations. *Proteins derived from the cytosol of the parent cell. The figure was prepared in part using BioRender.com, including crystal structures from the following references Sopkova et al. (1993), Ding et al. (2018), Kitchen et al. (2015), Makyio et al. (2012), and the PDB accession ID: 2CRN (unpublished).

The consensus on EV composition is widely debated and constantly evolving (Kowal et al., 2016; Théry et al., 2018; Jeppesen et al., 2019) (Figure 4). EVs have a modal diameter of 50–100 nm depending on the preparation method employed, with the vast majority of EVs being <200 nm in diameter independent of the method (Bachurski et al., 2019; Jeppesen et al., 2019; Ji et al., 2019; Brennan et al., 2020). EVs generally carry a slightly negative surface charge as indicated by zeta-potential estimations. The zeta-potential is a physical property of colloidal solutions which can be thought of as the potential difference between a solvent and the surface of solute particles. Larger absolute zeta-potential values indicate greater stability of the colloid. With regard to EVs, a slightly negative zeta-potential suggests that EVs exhibit sub-par stability behaviors in biological colloids and therefore are more likely than lipoproteins to interact with cell surfaces and other particles (Lan et al., 2011; Wang and Reed, 2011; Kozak et al., 2012; Deregibus et al., 2016; Vogel et al., 2017; Heath et al., 2018; Brown et al., 2020). EVs have masses in the megadalton range (Brown et al., 2020), or about 0.25 pg on average (Stratton et al., 2014). Most EVs are identified biochemically by the presence of certain proteins within their membrane (Théry et al., 2018). Pegtel and Gould provide further review of EV protein composition (Pegtel and Gould, 2019). Particularly, CD9, CD63, and CD81 are the most widely employed identifiers and any one of these tetraspannins are present in about five copies per EV. However, even among these markers there is known controversy. In one study, CD9 and CD81 were consistent EV markers, while CD63 expression depended on the experimental parameters (Yoshioka et al., 2013). Only about 30% of EVs coexpress two of these three tetraspannins (Tian et al., 2018). Current evidence suggests that a given cell only expresses certain EV subtypes even when considering these common EV identifiers. For example, about a quarter of cells derived from a single cell line secreted CD9+CD63+ EVs (Ji et al., 2019).

Contamination is a significant problem when evaluating EV composition (Figure 4). Using high resolution iodixanol density gradient centrifugation, Jeppesen et al. identified that ten of the top 25 proteins commonly associated with EVs were in fact correlated with protein contaminants and not EVs (Jeppesen et al., 2019). Furthermore, of the most abundant micro-RNAs identified, many were associated with non-vesicular purified fractions and not with small EVs. RNA binding proteins previously reported to be EV-correlated were also found in the high density, non-vesicular fraction. The Argonaute proteins involved in micro-RNA biogenesis were associated with non-vesicular particles, and not small EVs, in accordance with the findings of Arroyo et al. (2011) and contrary to Melo et al. (2014), implying that cells must secrete Argonaute proteins independently of small EVs. However, there is substantial controversy as to whether Argonautes are secreted independently and/or within EVs (Weaver and Patton, 2020). Additional recent experiments related to Argonaute have shown evidence that certain extracellular micro-RNAs may be actively regulated in their secretion and can be packaged either in Argonautes or EVs. Biró et al. (2019) showed that trophoblast cells of one type predominantly secreted EVs containing unbound miR-210, while trophoblasts of another type predominantly secreted Argonaute-bound miR-210 (Biró et al., 2019). Other experimental systems have also pointed to switch-like and hypoxia-inducible behaviors of miR-210 related to Argonautes (Noman et al., 2012; Hale et al., 2014). Given that Argonaute-bound micro-RNAs have unique cellular uptake mechanisms, the idea of regulating RNA function based on packaging the RNA within a specific carrier type is supported (Prud'homme et al., 2016). In investigating other RNA-binding proteins, such as the Major Vault Protein, a constituent of Vault complexes (Van Zon et al., 2003), Jeppesen et al. (2019) found no correlation of Vaults with small EVs, also contrary to previous reports (Teng et al., 2017). The findings by Jeppesen et al. further suggest that DNA is released through an amphisome-dependent mechanism that is generally exclusive of EVs <150 nm (Jeppesen et al., 2019) raising to question whether only EVs of a certain type can carry DNA. Overall, the latest technologies and findings point to a need for reassessment of EV composition especially in the context of proteins.

The RNA profile of EVs is being newly explored as a means to classify EVs contributions within complex biofluids without physical separation of the fluid constituents. Recent computational analyses suggest that there are six high-level extracellular cargo types when classified by RNA biotype distribution: two EV-related, one lipoprotein-related, and three RNA binding protein-related (Jeppesen et al., 2019). By comparing RNA-seq data from high-density vesicles, low-density vesicles, and high-density lipoproteins across two different studies (Lässer et al., 2017; Vickers, 2018), a set of 81 informative non-coding RNAs were identified that showed consistent differences in expression levels between carrier types vs. random sets. Then, by adapting a deconvolution algorithm previously used to estimate cell types and compositions within heterogeneous breast tumors (Onuchic et al., 2016), Murillo et al. estimated the number and composition of distinct cargo types existing within a biofluid using the set of 81 informative non-coding RNAs. RNA-seq analyses from 21 datasets consisting of 2,138 samples, each representing a single disease state for a single biofluid, were subjected to the deconvolution algorithm and resulted in 75 cargo profiles. Pairwise correlations between the 75 resulting cargo profiles and hierarchical clustering resulted in six high-level cargo types that were then verified experimentally by iodixanol cushioned density gradient ultracentrifugation. The computational deconvolution of cargo types including EVs from total extracellular RNA can help uncover biological insights that might have otherwise been missed because of the large variance in total extracellular RNA contents (Murillo et al., 2019). For example, 36 new micro-RNAs were identified as differentially expressed during exercise, 11 of which correlated with EV compartments and were related to striated muscle contraction pathways (Shah et al., 2017b; Murillo et al., 2019). In the future, we anticipate seeing a similar computational approach which can use RNA signatures to identify the magnitude that EVs from a given cell type are represented within a complex biofluid. We should soon be equipped with a greater toolkit to identify RNA signatures within EVs given recent focuses of the ERC program to develop robust experimental methods for EV preparation and characterization (Ainsztein et al., 2015; Das et al., 2019)^7^. Principally, there is a deficiency in computational characterization of EVs by their composition and we expect to see significant growth in this area.

The lipid composition of EVs is relatively understudied compared to RNAs and proteins as evidenced by H-indices^8^ of 132^9^, 62^10^, and 27^11^ for primary articles related to RNA, protein, and lipid composition respectively. Hundreds of lipid species have been identified in EVs across numerous accounts; cholesterol, phosphatidylcholine, and sphingomyelin derivatives are among the most common lipid components of EVs studied (by mol percent of total lipids). EVs tend to have a higher lipid:protein ratio than cells, but a smaller ratio than lipoproteins (Llorente et al., 2013; Lydic et al., 2015; Haraszti et al., 2016; Skotland et al., 2017a; Sun et al., 2019). Some reports indicate that EVs have different lipid contents than their parent cells (Subra et al., 2007; Lydic et al., 2015). These phenomena could present a potential mechanism of cell membrane homeostasis, or modulation of the recipient cell membrane. EVs from different cell types have a distinct composition (Skotland et al., 2017b, 2019). Furthermore, lipid signaling has been correlated with EV release and changes in EV composition (Phuyal et al., 2015; Hirsova et al., 2016; Skotland et al., 2019). *In vitro* experiments have indicated that normal vs. tumorigenic cells release EVs with differential lipid compositions (Brzozowski et al., 2018). *In vivo*, EV lipid composition can identify diseased patients vs. healthy controls (Skotland et al., 2017a; Tao et al., 2019). Principally, since EVs ~50 nm in diameter are ~50% lipid bilayer by volume (assuming a spherical vesicle, and uniform 5 nm membrane thickness) it is important to consider lipids holistically among other agents of EV functions. For example, pharmacologic studies have shown that phospholipids can have an affinity for G-protein coupled receptors and allosterically regulate their activity, hence even lipids of low relative abundance can elicit notable physiologic effects (Dawaliby et al., 2016). According to these findings, a deeper investigation into the lipid composition of EVs can yield important discoveries about both basic and translational EV biology.

EVs are also known to contain small-molecule metabolites (Figure 4) with purported biological activities. Organic acids, amino acids, fatty acids, and sugars have all been identified within EVs (Vallabhaneni et al., 2015; Brzozowski et al., 2018; Tao et al., 2019; Eylem et al., 2020). A multi-omic analysis found several metabolic pathways enriched in colorectal cancer-derived EVs, including aminoacyl-tRNA biosynthesis, nitrogen metabolism, and amino acid metabolism (Eylem et al., 2020). Notably, cancer-derived EVs could metabolically reprogram recipient cancer cells to incite the Warburg effect (Heiden et al., 2009; Zhao et al., 2016; Zhang Q. et al., 2018; Meng et al., 2019), and stem cell-derived EVs were metabolically tumor-supportive in a similar fashion (Vallabhaneni et al., 2015). Together, these studies point to EVs carrying and delivering raw materials that influence cellular metabolism at a number of pathway entry points. The study of EV-derived metabolites both *in vivo* and *in vitro* has thus far proved challenging due to the raw quantity of EVs required to surpass MS detection limits. However, a new procedure involving isotope labeling prior to MS has demonstrated detection of thousands of metabolites in only 2 mL of human serum starting material (Luo et al., 2018). Given these and other advancements in metabolomics, we can reach toward a deeper profiling of EVs in both healthy and disease states *in vivo*.

### 4.1. The Diverse RNA Contents of EVs

There have been significant research efforts toward understanding RNA content within EVs due to several reasons including:

1. The fact that EVs protect RNAs from degradation in the extracellular environment (Arroyo et al., 2011; Shurtleff et al., 2017; Pua et al., 2019).
2. The notion that EVs can be separated from other components of said biofluid, and concentrated to desirable levels for downstream analytics (Liu F. et al., 2017; Gyuris et al., 2019).
3. That RNAs contained within EVs encode biological state-specific information (Konstantinidou et al., 2016; Shah et al., 2017b; Murillo et al., 2019; Pua et al., 2019).
4. The hypothesis that EVs are intercellular communication agents that functionally transfer biological information between cells and tissues (Mathieu et al., 2019; Stahl and Raposo, 2019).
5. The great infrastructure to measure RNAs such as RNA-seq (Mardis, 2008; McCombie et al., 2019).

As we discussed in section 2, EVs' functional abilities, and more specifically their abilities to functionally transfer their RNA contents is a highly controversial topic. We find that the challenges in assessing EV-derived RNA functions arise from two primary questions: whether the study directly show that EV cargo delivery elicited a change in activity, and whether that activity change RNA-dependent. First, of the studies which show RNAs to be transferred between cells, many do not directly show a change in cellular activities (e.g., protein translation in the cell). For representative examples, see Ratajczak et al. (2006) and Valadi et al. (2007). The scientific community generally assumes that RNAs are active in some fashion after being transferred. Second, EVs have many other cargoes and therefore an RNA-dependent activity cannot be assumed. Additional experiments are needed to show that RNAs are the primary functional agent in a specific context. Throughout this review, we mainly discuss EVs' functional possibilities since the majority of published literature currently points in that direction. However, it is important to note these challenges and limitations as an opportunity for new investigations. Based on our biophysical and biochemical review of EVs we find it unlikely that the RNA contents of EVs are alone sufficient to elicit many of the EV-related phenomena discussed here.

Extracellular RNAs in human serum are present in ng/mL concentrations and predominantly in the 20–200 nt size range (Zhou Z. et al., 2019). It should be noted that there are a number of non-vesicular extracellular RNA carriers such as lipoproteins (Vickers et al., 2011), RNA-binding proteins (Arroyo et al., 2011), and exomeres (Zhang Q. et al., 2019). Current evidence suggests that EVs contain approximately half of circulating RNAs in plasma (Wei et al., 2017; Karimi et al., 2018). EVs have been correlated with numerous coding and non-coding RNA biotypes. Here we will discuss miRNA, tRNA, yRNA, circRNA, and lncRNA though several other RNA types are present (Freedman et al., 2016; Godoy et al., 2018).

#### 4.1.1. mRNAs

mRNAs have been widely reported as EV cargo, and there is still much to study with regard to their functions when packaged within EVs *in vivo*. Possible mRNA functions aside from providing a template for protein production (mRNA translation) include riboswitch systems, ribozyme activities, cis-regulatory mRNA sequence presentation or concealment, and trans-regulatory behaviors, which are all driven by mRNA folding and/or processing (Wachter, 2014). Early studies have indicated that EV-related mRNAs are most likely fragmented and that untranslated regions are enriched within EVs (Nolte'T Hoen et al., 2012; Batagov and Kurochkin, 2013), supporting the idea that EVs can facilitate the many possible mRNA regulatory roles described by Wachter (2014). EV-related mRNA content has also been correlated with disease and disease progression. Shao et al. (2015) showed that methylguanine DNA methyltransferase and alkylpurine-DNA-N-glycosylase mRNA levels correlated with responses to temozolomide treatment in glioblastoma patients (Shao et al., 2015). As discussed in more detail in section 2, (Hyenne et al., 2019; Verweij et al., 2019) have recently shown *in vivo* evidence to support that EVs can travel to distant tissues and interact with resident macrophages. The EV-immune system interaction directly supports an immune signaling hypothesis and broader role for mRNA content within EVs, insofar as EV-derived mRNAs could be either passively communicating with, or actively influencing immune cells (Hyenne et al., 2019; Verweij et al., 2019). Our review identified strong evidence and techniques showing functional mRNA transfer in limited instances. Some groups have demonstrated functional mRNA transfer by EVs using Cre-LoxP systems (Lewandoski, 2001; Ridder et al., 2015; Zomer et al., 2015, 2016). For example, Zomer et al. (2015) showed that cellular co-culture of a Cre-expressing cell type with a reporter cell type induced GFP expression in reporter cells by transfer of Cre mRNA and successful Cre-directed recombination (Zomer et al., 2015). However, others have acknowledged that with this Cre-LoxP system it is unclear whether successful recombination is attributable to Cre protein transfer vs. Cre mRNA transfer by EVs (de Jong et al., 2020). Furthermore, a luciferase-mRNA reporter construct was incapable of functional mRNA delivery in both small and large EVs (Kanada et al., 2015). There is, therefore, much remaining to investigate to clarify the role of EV-derived mRNAs and their potential functional mechanisms.

#### 4.1.2. micro-RNAs

Micro-RNAs (miRNAs) are the most heavily studied and strongly characterized extracellular RNA biotype contained within EVs. Hundreds of miRNA species can be detected in a given biofluid and there is longitudinal evidence that each fluid type has a distinct miRNA composition which can be changed by health status including pregnancy, urothelial cancers, neurological disorders, diet, and exercise (Weber et al., 2010; Rome, 2015; Konstantinidou et al., 2016; Lusardi et al., 2017; Shah et al., 2017b). miRNAs are ~20 nucleotide long and can modulate gene expression by mRNA silencing, translational activation, and even more complex intra-nuclear functions. For recent, in-depth reviews on miRNA biogenesis and mechanisms of action (see O'Brien et al., 2018; Gebert and MacRae, 2019; Trabucchi, 2019). There are currently 2,656 annotated human miRNAs with targets, having 1,610,510 gene targets and 29,161 unique gene targets in the miRDB (release 6.0, mid-2019) (Chen and Wang, 2019; Liu and Wang, 2019) indicating that each miRNA has a mean of 606 gene targets. For example, the miRDB database predicts 1,408 targets of mir-181b-5p and 15 targets with a very-high confidence score^12^. Therefore, it can be difficult to ascribe specific roles to a given miRNA. However, because many miRNAs exhibit tissue-specific and state-specific expression patterns, the interest to use miRNAs as biomarkers has grown significantly over the past ten years. As noted in other sections, reports as early as 2007 have argued that cells package miRNAs within EVs and that they are transferable to other cells with functional consequences, which coined the term “exosomal shuttle RNA” (Valadi et al., 2007). More recently, a CRISPR-Cas9 reporter system was designed to track guide-RNA (miRNA-like) transfer through EVs. Interestingly, only 0.2% of cells were converted into reporter cells by co-culture with Cas9-expressing cells, indicating that EV-packaged small RNAs alone face a substantial pharmacological challenge to elicit a biological effect (de Jong et al., 2020). Other studies also support the pharmacological weakness of miRNA-like species packaged within EVs. Reshke et al. (2020) showed that an siRNA construct using a pre-miRNA backbone sequence, which tends to be enriched in EVs, could increase siRNA loading up to one copy per EV (58- to 7,000-fold increase depending on the cell type) and thereby drastically increase EV potenty *in vivo* (Reshke et al., 2020). Furthermore, reports that EVs package and protect miRNAs in human extracellular fluids has encouraged development of miRNA-based liquid biopsies for high-risk clinical applications such as oncology where frequent sampling can provide crucial insights into treatment decisions and disease progression. Interestingly, numerous exogenous miRNAs circulate in human plasma from dietary sources including plants, milk, and egg products although it is unclear to what extent they are packaged within EVs (Baier et al., 2014; Zempleni et al., 2015) and whether dietary-derived small RNAs are functionally active (Chan and Snow, 2017; Dávalos et al., 2019). Organized miRNA loading into tumor-derived EVs, and cell-free pre-miRNA processing by EVs have also been described (Melo et al., 2014; Cha et al., 2015; Clancy et al., 2019) which suggests a special relationship between EVs and miRNA biology. Although miRNAs have been heavily studied, there remains much to be discovered in the context of EV biology.

#### 4.1.3. tRNA-Derived Small RNAs

tRNA-derived small RNA fragments (tDRs) are a key component of EV-derived RNAs, taking large fractions of the RNA in most biofluids tested (Godoy et al., 2018; Sork et al., 2018). tDR production has been related to the enzymes RNase P, Angiogenin, Dicer, and Elac2 (Liapi et al., 2019). The ratio of miRNA to tDRs varies largely between biofluids, ranging between 0.004 and 72 in bile and plasma, respectively (Godoy et al., 2018). tDRs exhibit extensive diversity due to there being over 500 human tRNA genes (though ~250 of these have been estimated to be transcriptionally inactive) (Torres, 2019; Torres et al., 2019) and at least five known fragmentation patterns (Godoy et al., 2018). Therefore, tDRs have a substantial contribution to the overall non-coding RNA landscape (Gebetsberger and Polacek, 2013) and should be matched with miRNAs in terms of their scope and significance (Liapi et al., 2019). tDRs are known to have regulatory roles during translation by binding ribosomal subunits and aminoacyl t-RNA synthetases (Mleczko et al., 2018), and more complex levels of regulation by binding argonaute and piwi proteins (Balatti et al., 2017). Accordingly, tDRs have been correlated with diverse and complex biological processes such as cardiomyocyte hypertrophy and skeletal muscle homeostasis (Liapi et al., 2019). Interestingly, T-cells appear to leverage tDRs secreted through EVs as a control structure to inhibit T-cell activation and cytokine release (Chiou et al., 2018). Given the increasing importance of tDRs, new and optimized annotation pipelines for tRNA fragments are becoming available to improve bioinformatic analyses (Shi et al., 2018). tDRs are an underappreciated component of EVs with vastly undiscovered biological roles and we anticipate a greater focus on them in future investigations.

#### 4.1.4. Y RNAs

Y-RNAs (~100 nt) are one of the most abundant RNA components of EVs, and specifically can dominate within blood (Nolte'T Hoen et al., 2012; Godoy et al., 2018). The human genome contains four annotated Y-RNA genes (1, 3, 4, and 5). Y-RNAs were originally discovered in association with the circulating ribonucleoprotein autoantigens Ro60 and La in individuals with Systemic Lupus Erythromatosus or Sjögren's disease. Stress management, DNA replication, and post-transcriptional gene regulation have all been correlated with Y-RNA complexes. There is preliminary evidence linking EV-bound Y-RNAs with immune system regulation by way of toll-like receptor signaling. Y-RNAs have an observed affinity to Ro60, La, nucleolin, and a number of functionally-annotated RNA-binding proteins (Sim and Wolin, 2011; Kowalski and Krude, 2015; Driedonks and Nolte-T'Hoen, 2019). In human blood plasma, a high percentage of reads produced by small RNA-seq (reported as high as 67%) map to Y-RNAs (Driedonks and Nolte-T'Hoen, 2019). Y-RNA fragments are found in most human biofluids, within which Y-RNA 4 accounts for the majority of total Y-RNA reads (Godoy et al., 2018). Y-RNA differential expression in human blood plasma has been linked to inflammatory signaling related to cardiovascular disease. Furthermore, acute maximal exercise on a treadmill significantly changes the Y-RNA expression profile and increases total Y-RNA expression. (Shah et al., 2017b). In a cell culture model of human glioblastoma, Y-RNAs were significantly different between extracellular compartments (exosomes, microvesicles, non-vesicular complexes) while micro-RNAs were not, indicative of a selective pressure on Y-RNAs (Wei et al., 2017). EV-derived Y-RNAs have a clear significance specifically with regard to inflammation and immune system homeostasis.

#### 4.1.5. circRNA and lncRNAs

Long non-coding RNA (lncRNA) and circular RNA (circRNA) species are generally present to lesser extents in circulating biofluids (Godoy et al., 2018), though their importance has been appreciated in a number of contexts and has been growing (Li et al., 2018c). circRNAs are abundant in the cellular nucleus and have been shown to positively regulate their parent genes by binding to polymerases (Zhang et al., 2013). Additionally, circRNAs like other non-coding RNAs, express tissue and developmental stage-specifically, and interact with miRNA pathways (Memczak et al., 2013; Salzman et al., 2013; Rybak-Wolf et al., 2014). Interestingly, in an effort to create an atlas of circRNA expression in the human body, many circRNAs were found in higher levels than linear RNAs from the same gene (Maass et al., 2017). lncRNAs, like circRNAs, have a largely unknown function beyond speculations of their regulatory roles. lncRNAs are strongly present in certain EV subsets such as high-density EVs released by mast cells (Lässer et al., 2017). Furthermore, colorectal cancer-cell derived EVs in some instances release high concentrations of specific lncRNAs with nuclear uptake by recipient cells (Hinger et al., 2018). With regard to EVs specifically, exoRBase currently hosts a collection of ~60,000 circRNAs and ~15,000 lncRNAs identified by RNA-seq of 92 human blood EV samples (Li et al., 2018c). We anticipate further developments in circRNA and lncRNA EV-related research.

## 5. Extracellular Vesicle Physiology and Biomedical Relevance

EVs are related to a number of physiological phenomena. Here we discuss more details on the roles of EVs as signal transducers and biomarkers for physiological states, how they can be leveraged by microbes to deepen host-microbe interactions, how EVs can be implemented as a novel therapeutic strategy, and provide a unique window into fetal health and development.

### 5.1. Extracellular Vesicle-Syndicated Physiological Processes

EVs are temporally and spatially regulated, and serve in signaling roles that direct biological processes. Current evidence suggests that EVs derived from human biofluids can provide a state-specific window into the biology of cells in closest proximity to the fluid. For example, the majority of plasma-derived EVs originate from platelets and erythrocytes (Karimi et al., 2018). Therefore, molecular profiling of blood-derived EVs should provide great detail about the hematologic system. Similarly, we would expect, and have found a number of reports that cerebrospinal fluid (Burgos et al., 2014; Lusardi et al., 2017), sweat (Wu and Liu, 2018), and urine derived EVs (Gonzales et al., 2009; Merchant et al., 2017) accurately picture the central nervous system, skin, and renal systems, respectively. Some of the earliest accounts of EVs were related to neural and connective tissue signaling (Anderson, 1969; Grillo, 1970; Dermietzel et al., 1972). Beyond Grillo's initial identification of EVs at mouse neuromuscular junctions (Grillo, 1970), EVs carry active Wnt proteins in both humans and *Drosophila* across neuromuscular junctions, thereby exerting a broad influence on developmental pathways (Korkut et al., 2009; Gross et al., 2012; Koles et al., 2012). EVs have been more narrowly characterized to coordinate central nervous system homeostasis by controlling synaptic activity, plasticity, myelination, and neuroprotective inflammation (Korkut et al., 2013; Budnik et al., 2016; Holm et al., 2018). In bone and associated connective tissues, EVs are crystal nucleation sites associated with both facilitation (Anderson, 1969; Ali et al., 1970; Hasegawa et al., 2017) and inhibition (Li et al., 2019) of mineralization. Solid-phase EVs, which are often called matrix vesicles, have also been located in extracellular matrices more generally, although they have an indeterminate biological role (Huleihel et al., 2016; Hussey et al., 2020). During exercise, organism-wide signaling cascades involved with the physical stress response are mediated by a variety of myokines and regulatory RNAs. It is thought that exercise-induced adaptations are largely mediated by this extracellular milieu (Adams and Bamman, 2012; Shah et al., 2017b; Whitham and Febbraio, 2019). EV secretion is upregulated during exercise and correlated with novel myokines (Whitham et al., 2018). Furthermore, there is some evidence to suggest that skeletal muscle can undergo hypertrophy responses, independently of muscle progenitor cell fusion, by way of satellite cell-derived EV signals (Mccarthy et al., 2011; Fry et al., 2017). Some groups are investigating the roles of stem-cell derived EVs in relation to the damage-induced muscle regeneration response (Mitchell et al., 2019), and more broadly as a homeostatic strategy (Riazifar et al., 2016). From this perspective, EVs could be considered agents that can integrate and translate complex signaling messages that are required to orchestrate processes among heterogeneous cell populations.

Many research efforts have focused on identifying EV-related biochemical signatures that can elucidate physiological details of diseased and healthy states, diagnose diseases, and predict clinical outcomes. In complex phenomena such as post-myocardial infarction ventricular remodeling, cardiomyocyte death recruits immune cells which can lead to either beneficial or adverse remodeling outcomes depending on specifics of the response (e.g., time to resolution, scarring, and other cellular factors). An RNA-seq study with 22 subjects identified 21 micro-RNA candidates that showed significant differential expression between those with beneficial vs. adverse remodeling outcomes. Top pathways targeted by these micro-RNAs include immune cell signaling, fibrosis, and apoptosis, indicating that specific inflammatory states lead to positive or negative resolutions of myocardial infarction (Danielson et al., 2018). Furthermore, these three pathways are unified by NF-κB signaling, which can explain the clinical utility of novel therapies such as the IL-1B antagonist, anakinra, in treating myocardial infarction (Abbate et al., 2013). A large-scale observation of participants in the Framingham Heart Study identified 16 miRNAs associated with insulin resistance and adiposity (Shah et al., 2017a). Because many biomarker studies examine exRNA more broadly, including those cited here, the findings are potentially EV-related but not specific. For more information on exRNA biology and the challenges related to EV-derived RNA studies see the reviews by Li et al. (2018a) and Mateescu et al. (2017). Studying EVs may provide a distinct advantage over a total extracellular RNA approach since EV-contained RNAs can undergo RNA-unshielding and thereby carry a higher functional potential than when protein-bound in circulation (Nabet et al., 2017). Furthermore, EVs have demonstrated extensibility to sample from diverse cellular compartments including the nucleus and mitochondria (Yokoi et al., 2019; Zhang W. et al., 2019). Yokoi et al. detected increased nuclear content (genomic DNA) loading into EVs within blood from ovarian cancer patients and in cell lines exposed to genotoxic drug treatment *in vitro* (Yokoi et al., 2019). EV-related biomarkers remain largely unexplored.

### 5.2. Host-Microbe Interactions

The physiological role of exogenous EVs is being newly investigated. EVs are secreted by most organisms and accordingly, given the extensive population of humans by microbial species and viruses, we can predict a substantial number of exogenous EVs to exist in human biofluids. A large proportion of circulating RNAs, including miRNAs, are contributed by the microbiome. In some EV preparations, 75% of the total RNA reads originate exogenously (Wang et al., 2012; Fritz et al., 2016; Galvanin et al., 2019). Tulkens et al. outlined a newly-developed combinatorial strategy including ultrafiltration, chromatography, and density gradient ultracentrifugation to fractionate bacterial- and host-derived EVs and other contaminating particles (Tulkens et al., 2020). This and other new experimental approaches we have reviewed, when carefully implemented, will help us gain a greater understanding of the mutualistic human-microbe relationship. Viruses also interact deeply with human physiology at a level not yet appreciated, and we find substantial evidence that viruses exert organism-level control over humans in part by leveraging EVs. HIV-infected T cells produced EVs by plasma membrane budding and scission from an endosome-like domain, showing that viruses can flex EV biogenesis pathways to their reproductive advantage (Booth et al., 2006). This study illustrates the gap between what we know, and what we think we know about EV physiology. More recent studies have shown HIV assembly by plasma membrane modulation (Gerber et al., 2015), and release of EVs carrying HIV-Nef and HIV-Env (Raymond et al., 2011; Arakelyan et al., 2017). EVs from HIV-infected cells were also shown to initiate DNA replication in recipient cells and bring infected cells out of latency. A multi-omic analysis of these EVs found a number of cyclin-dependent and receptor tyrosine kinases that could be responsible for the effects (Barclay et al., 2019). A small subset of HIV-infected individuals, called elite controllers, can control the viral load without antiretroviral drug regimens. Notably, elite controllers exhibited differential expression of several circulating miRNAs, including miRNA-223, which have been known to affect HIV latency (Narla et al., 2018) indicating a possible role of EVs in controlling viral replication. EVs can also package virally-derived, functionally-implicated miRNAs. EBV-infected B cells released EVs that contained miRNAs, and downregulated genes which when repressed were correlated with EBV-related disease (Pegtel et al., 2010). Interestingly, retrovirus-leveraged mechanisms related to EV biogenesis and transport appear evolutionarily integrated in mammals. Arc, a protein-coding gene expressed by neurons, is a synaptic plasticity controller that can be packaged within EVs in a capsid structure and transferred between cells (Pastuzyn et al., 2018). Furthermore, highly-active LINE-1 retrotransposons contribute to genetic diversity (Beck et al., 2010) and could horizontally-transfer *in vitro* by way of EVs (Balaj et al., 2011; Kawamura et al., 2019). Together, these reports emphasize the continued need to study EV physiology in diverse biological contexts and particularly with a focus on host-microbe interactions.

### 5.3. Novel Therapeutic Strategies

EVs are particularly interesting for novel therapeutic strategies. Because EVs have a suspected mechanism for targeting and delivering their cargoes to certain cell populations (Hyenne et al., 2019; Verweij et al., 2019), several groups are investigating EV-syndicated pharmacologic strategies to effectively increase drug potency and decrease systemic toxicity (De Jong et al., 2019; Kanada et al., 2019). Kanada et al. showed that minicircle DNA encoding a prodrug converting enzyme was delivered by EVs to tumors *in vivo*, and prodrug administration led to tumor death when at least 1% of the tumor cells in a given tumor received the minicircle DNA. Dysregulated EV signaling has also been implicated in tumor immunology (Ricklefs et al., 2018) and could uncover additional pharmacologic opportunities. The mechanisms which control EV trafficking and cargo transfer are still largely unclear (Russell et al., 2019) and will challenge therapeutic efforts. Early studies have described increased EV release and uptake under acidic conditions, which is a hallmark of the tumor microenvironment, and elucidated the complexity of EV trafficking (Heiden et al., 2009; Parolini et al., 2009).

### 5.4. Fetal Diagnostics

There are a number of clinical scenarios when it may be desirable to attain the genetic information of a developing fetus, such as when the pregnancy is at-risk for yielding a trisomy or other chromosomal abnormalities^13^. Most notably, with a maternal age greater than 35, the prevalence of fetal trisomy 21 development is significantly higher and disease prevalences continue to increase with maternal age^14^. The primary techniques currently employed to study fetal cfDNA include qPCR, and DNA sequencing, where the samples are obtained from maternal blood. Fetal DNA contributions to total cell-free DNA range between 6 and 25% during the three trimesters of pregnancy, with the percentage directly related to developmental stage (Fan et al., 2012). Current fetal cfDNA tests have reported positive prediction rates of 97% in identifying several trisomies (Chen et al., 2011). Cell-free DNA testing is limited by: a lack of cost-effectiveness studies, and poor quality outside of identifying abnormalities other than trisomies 13, 18, and 21 (Gekas et al., 2014).

Deep and high-coverage sequencing studies of the fetal exome and genome have demonstrated the potential of extracellular RNA to make clinical assessments of the fetal genome at various time points (Fan et al., 2012). Recent studies affiliated with the sequencing company BGI Genomics have indicated that low-coverage (0.25×) sequencing of EV-derived DNA from only 250 μL of maternal blood plasma can cover all chromosomes, and notably, provided twice as much sampling of the mitochondrial genome than a cell-free approach (Zhang W. et al., 2019). Given the differential expression of genes within EVs vs. cell-free fluids, we believe that there is considerable potential for an EV-based approach for prenatal clinical assessment.

## 6. Databases

There are a number of online public databases which host EV-related gene expression data and serve as a reference for the research community (Table 1). Vesiclepedia hosts a community-contributed catalog of mRNAs, miRNAs, proteins, lipids, and metabolites which have correlated with EVs (Pathan et al., 2019)^15^. Most samples are linked to a PubMed ID where users can find the published study. A majority of the samples are human-derived, however, the database is not exclusive to human studies. ExoCarta is a similar resource as Vesiclepedia, but the catalog is exclusive to small EV studies (Keerthikumar et al., 2016)^16^. Vesiclepedia and Exocarta are helpful for finding which molecular signatures have been previously identified in a specific EV population. There are not yet any databases for single-EV or single-cell EV data that we are aware of, but in the near future we expect to see more. GTEx, the genotype-tissue expression database, was an NIH Commonfund program which collected over 17,000 samples from 54 human tissues and 948 donors. The majority of samples underwent both genotyping and RNA sequencing, thereby establishing an extensive atlas of gene expression by tissue type (Lonsdale et al., 2013; Mele et al., 2015)^17^. This dataset contains foundational information to begin developing a correlation between extracellular RNA and tissue type i.e., to determine how each tissue contributes to the overall extracellular RNA landscape. ExoRBase contains RNA-seq datasets analyzing circular RNA, messenger RNA, and long non-coding RNA expression from human blood-derived small EVs (both healthy and disease states). ExoRBase leverages GTEx annotations to predict a tissue type and a tissue specificity score for each gene entry (Li et al., 2018c)^18^. EV-TRACK (transparent reporting and centralizing knowledge in extracellular vesicle research) is a resource which tabulates the methodologies of published EV studies (Van Deun et al., 2017)^19^. An investigator can easily sort through indexed experiments by metrics such as biofluid type, separation method, and analysis methods. As a result, EV-TRACK is helpful to identify subsets of EV studies and their quality. For example, with the search strategy “Study aim: ‘Omics’ AND Species: ‘Homo sapiens’” we were able to identify 139 publications, 64 of which studied whole human biofluids (i.e., not cell culture-derived)^20^.

**Table 1:** Databases related to extracellular vesicle composition.

**Name**	**URL**	**Content**	**EV focused?**	**Humans only?**	**Studies**
Vesiclepedia	microvesicles.org	Protein, mRNA, miRNA, lipids/metabolites	Yes	No	1,254
ExoCarta	exocarta.org	Protein, mRNA, miRNA, lipids/metabolites	Yes	No	286
GTEx	gtexportal.org	DNA-seq, RNA-seq	No	Yes	948^*^
ExoRBase	exorbase.org/exoRBase/toIndex	circRNA, mRNA, lncRNA	Yes	Yes	92
EV-TRACK	evtrack.org	experimental database	Yes	No	2,194
exRNA Atlas	exrna-atlas.org	RNA-seq, qPCR	No	Yes	36

* *In a single, multi-center study, 948 unique donors contributed 17,382 tissue samples*.

To address the ERC program goal of generating an exRNA reference catalog, 2,270 extracellular small RNA-seq and 3,039 qPCR profiles across 19 studies and 23 health states were compiled in the ERC Consortium data repository, the exRNA Atlas (Murillo et al., 2019)^21^. The thousands of RNA-seq profiles currently hosted on the Extracellular RNA Atlas encompass samples from a variety of human biofluids and health conditions. In most cases, total cell-free biofluid RNA, to which EVs are a major contributor, was isolated from one of five biofluids (cerebrospinal fluid, plasma, saliva, serum, and urine) and prepared for sequencing using commercially available kits (Murillo et al., 2019). The data were uniformly processed and quality-controlled by the extracellular RNA processing toolkit (exceRpt) pipeline built by ERC investigators (Rozowsky et al., 2019). The exRNA Atlas has an extensive graphical interface where users can sort through samples by condition (health status), biofluid, RNA source, and RNA isolation kit. The exRNA Atlas can also host and analyze user-uploaded datasets with a variety of tools including differential expression by DESeq2 (Love et al., 2014), WikiPathways custom pathway queries (Slenter et al., 2018), principle component analysis (PCA) (Shlens, 2014), dimensionality reduction by t-stochastic neighborhood embedding (van der Maaten and Hinton, 2008), and computational deconvolution of extracellular cargoes by XDec (Murillo et al., 2019). These databases are continually expanding and are the foundations of a comprehensive atlas of RNA contents in EVs.

## 7. Discussion

In this review we have provided historical context for EVs and their rise to prominence in biomedical research, reviewed EV biogenesis and fates with perspective on functional outcomes, and identified some of the latest experimental approaches for preparing and analyzing EVs with a focus on clinical applications. We also discussed the physiology and biochemical content of EVs with a focus on RNA biotypes, and provided an overview of online databases hosting EV-related experimental findings. The research community has consistently advocated for the importance of EVs especially with regard to their RNA contents. EVs are hypothesized to act as intercellular communication agents that functionally transfer biological information between cells and tissues. EVs protect RNAs from degradation in the extracellular environment. Furthermore, RNAs contained within EVs encode biological state-specific information, and RNA-containing EVs can be easily and frequently collected in a clinical setting and analyzed. In the future, it is possible to envision noninvasive EV-based diagnostics enabling precision health applications–where sampling an individual over time is beneficial to gain insight into the evolution of a dynamic health or disease state (Chen et al., 2012). However, stoichiometrically, and based on the principles of mass action-driven biochemical reactivity, it is difficult to conclude that the RNA contents of EVs alone are sufficient to elicit EV-related phenomena (Chevillet et al., 2014; Wei et al., 2017; He et al., 2019). We therefore advocate, as others have, for a holistic study of EVs (Soekmadji et al., 2018). We propose, due to significant advancements in omics technologies (both hardware and software pipelines), a greater implementation of existing multi-omics strategies (Chen et al., 2012; Vallabhaneni et al., 2015; Barclay et al., 2019; Eylem et al., 2020) to characterize EVs in a detailed and integrative fashion (Domanskyi et al., 2019). Omics technologies are continuing to uncover and describe distinct EV subtypes in specialized biological contexts. Stephen Badylak's group at Pittsburg has provided numerous accounts of matrix-bound nanovesicles, which are solid-phase EVs deposited into the extracellular matrix rather than into a liquid-phase (Huleihel et al., 2016; Hussey et al., 2020).

As we indicated throughout this review, there is a concerted effort toward single-EV characterization and a number of projects under the ERC commonfund have recently shifted to new stages^22^. Importantly, we have also indicated a need to comprehensively characterize the EV secretome by cell and tissue type, i.e., the formation of an atlas which describes the profile of EVs that each cell type secretes under normal and abnormal conditions. The importance of accounting for biological diversity when examining EV biology cannot be understated. Cell specialization, cell cycle stage, stress, infection, inflammation, and developmental programs, among other factors which we have not yet identified, can all potentially influence observed biological and physiological phenomena related to EVs and their biochemical contents. After such an atlas has been created, we can clarify our understanding of the EV secretome within a particular biofluid and draw clinically relevant conclusions.

## Author Contributions

EV wrote and reviewed the manuscript and generated the figures. GM conceived of and supervised the project, reviewed, and edited the manuscript. All authors listed have made a substantial, direct and intellectual contribution to the work, and approved it for publication.

## Conflict of Interest

EV declares equity ownership in Gilead Sciences, Inc., an American biotechnology company. The remaining author declares that the research was conducted in the absence of any commercial or financial relationships that could be construed as a potential conflict of interest.

## Footnotes

^1^http://apps.webofknowledge.com/ (accessed February 29, 2020).

^2^https://systembio.com/shop/exoquick-ultra-ev-isolation-kit-serum-plasma/ (accessed February 23, 2020).

^3^https://www.fluigent.com/microfluidic-expertise/what-is-microfluidic/microfluidic-definitions-and-advantages/ (accessed February 20, 2020).

^4^https://www.particle-metrix.de/en/products/zetaview-nanoparticle-tracking (accessed March 24, 2020).

^5^https://izon.com/qnano-gold/ (accessed March 28, 2020).

^6^https://clinicalkey.com (accessed March 5, 2020.

^7^https://commonfund.nih.gov/exrna/fundedresearch (accessed April 11, 2020).

^8^https://apps.webofknowledge.com citation report generated by advanced search, and then refined by articles for the document type (accessed April 10, 2020).

^9^RNA: TS=[“transcript*” AND (“extracellular vesicle” OR “exosome*” OR “microvesicle*”)].

^10^proteins: TS=[“proteome*” AND (“extracellular vesicle” OR “exosome*” OR “microvesicle*”)].

^11^lipids: TS=[(“lipid composition” OR “lipidome*” OR “lipid profile”) AND (“extracellular vesicle” OR “exosome*” OR “microvesicle*”)].

^12^http://www.mirdb.org (accessed April 6, 2020).

^13^https://www.mayoclinic.org/tests-procedures/noninvasive-prenatal-testing/about/pac-20384574.

^14^https://www.dynamed.com (accessed April 6, 2020).

^15^http://microvesicles.org/

^16^http://www.exocarta.org/

^17^https://www.gtexportal.org/home/

^18^http://www.exorbase.org/exoRBase/toIndex

^19^http://evtrack.org/

^20^evtrack.org (accessed January 28, 2020).

^21^exrna-atlas.org

^22^https://commonfund.nih.gov/exrna/fundedresearch (accessed April 11, 2020).
